# MXene-Derived Defect-Rich TiO_2_@rGO as High-Rate Anodes for Full Na Ion Batteries and Capacitors

**DOI:** 10.1007/s40820-020-00471-9

**Published:** 2020-06-16

**Authors:** Yongzheng Fang, Yingying Zhang, Chenxu Miao, Kai Zhu, Yong Chen, Fei Du, Jinling Yin, Ke Ye, Kui Cheng, Jun Yan, Guiling Wang, Dianxue Cao

**Affiliations:** 1 Key Laboratory of Superlight Materials and Surface Technology (Ministry of Education), College of Material Science and Chemical Engineering, Harbin Engineering University, Harbin, 150001 People’s Republic of China; 2 State Key Laboratory of Marine Resource Utilization in South China Sea, Hainan Provincial Key Laboratory of Research on Utilization of Si-Zr-Ti Resources, College of Materials Science and Engineering, Hainan University, 58 Renmin Road, Haikou, 570228 People’s Republic of China; 3 Key Laboratory of Physics and Technology for Advanced Batteries (Ministry of Education), College of Physics, Jilin University, Changchun, 130012 People’s Republic of China

**Keywords:** MXene–Ti_2_CT_*x*_, Vacancy oxygen, Self-supporting, TiO_2_ anodes, Sodium ion battery and capacitor

## Abstract

Sodium ion batteries and capacitors have demonstrated their potential applications for next-generation low-cost energy storage devices. These devices's rate ability is determined by the fast sodium ion storage behavior in electrode materials. Herein, a defective TiO_2_@reduced graphene oxide (M-TiO_2_@rGO) self-supporting foam electrode is constructed via a facile MXene decomposition and graphene oxide self-assembling process. The employment of the MXene parent phase exhibits distinctive advantages, enabling defect engineering, nanoengineering, and fluorine-doped metal oxides. As a result, the M-TiO_2_@rGO electrode shows a pseudocapacitance-dominated hybrid sodium storage mechanism. The pseudocapacitance-dominated process leads to high capacity, remarkable rate ability, and superior cycling performance. Significantly, an M-TiO_2_@rGO//Na_3_V_2_(PO_4_)_3_ sodium full cell and an M-TiO_2_@rGO//HPAC sodium ion capacitor are fabricated to demonstrate the promising application of M-TiO_2_@rGO. The sodium ion battery presents a capacity of 177.1 mAh g^−1^ at 500 mA g^−1^ and capacity retention of 74% after 200 cycles. The sodium ion capacitor delivers a maximum energy density of 101.2 Wh kg^−1^ and a maximum power density of 10,103.7 W kg^−1^. At 1.0 A g^−1^, it displays an energy retention of 84.7% after 10,000 cycles.

**Electronic supplementary material:**

The online version of this article (10.1007/s40820-020-00471-9) contains supplementary material, which is available to authorized users.

## Highlights

- A freestanding MXene-derived defect-rich TiO_2_@reduced graphene oxides (M-TiO_2_@rGO) foam electrode was fabricated.
- M-TiO_2_@rGO presents fast Na^+^ storage kinetics due to capacitive contribution.
- M-TiO_2_@rGO foam electrode displays a capacity retention of 90.7% after 5000 cycles.

**Electronic supplementary material:**

The online version of this article (10.1007/s40820-020-00471-9) contains supplementary material, which is available to authorized users.

## Graphical abstract

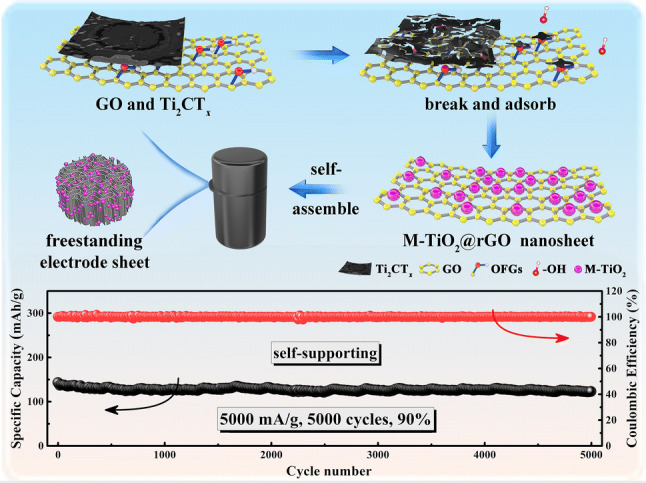

## Introduction

With the high-speed development of electric vehicles and smart grids, the market for electrochemical energy storage devices (EESDs) is bound to expand rapidly [1]. Due to the shortage of lithium resources, lithium-ion batteries are facing the difficulties of increasing cost and future availability [2]. Therefore, sodium ion batteries (SIBs) have attracted great interest due to their low cost and high sodium abundance [3, 4]. To date, Na_3_V_2_(PO_4_)_3_ with sodium super ionic conductor (NASICON) structure has been regarded as one of the most promising cathodes [5, 6]. Although NASICON-type cathodes promise fast sodium ion diffusion and high-power density, the rate performance of sodium ion full cells is hindered by the anode materials. Recently, a hybrid device called sodium ion capacitor (SIC) [7, 8] has offered another choice for EESDs, owing to its comprehensive advantages of high energy and power densities. Usually, a SIC consists of a battery-type anode and a capacitor-type cathode to bridge the strengths of SIBs and supercapacitors [9]. The key to constructing a high-performance SIC is to couple a suitable anode with a cathode, wherein they need well match in rate abilities. Thus, anode materials with fast sodium storage kinetics have become a key limitation in developing superior rate performance SIBs and SICs compared to fast energy storage cathodes.

According to the working principles, anode materials can be divided into intercalation, alloying, and conversion types [10, 11]. Usually, intercalation-type anodes avoid damaging volume expansion and contraction, which is a perplexing problem in alloying and conversion-type anodes [12]. Thus, various intercalation-type anodes have been explored for SIBs, such as hard carbon [13], layered titanium-based oxides [14], and molybdenum-based material [10]. Among these, TiO_2_ is considered to be one of the most promising SIB anode materials due to its safety and low work potential relative to the Na^+^/Na redox couple [15, 16]. However, its electrochemical performance is severely restricted by poor electron mobility (~ 10^−12^ S cm^−1^) and low sodium ion diffusivity [9, 17]. Defect engineering plays an important role in improving the physical and chemical properties of metal oxides. Oxygen vacancies have recently been shown to be able to significantly enhance the electronic and ionic conductivity of TiO_2_, thus enhancing the ions storage capacity and catalytic performance [18–20]. Theoretical calculations also indicate that oxygen vacancies can increase the number of reactive sites, decrease the reaction barrier, and improve the ionic diffusion path [19, 21]. Thus, it is an effective approach to improve the properties of TiO_2_. Another strategy, preparing nanomaterials has presented a unique advantage in shortening the sodium ion transport pathway [9, 22]. Ti-based MXene materials have been reported as a capable precursor for producing nano-Ti-based oxides and related composites owing to its two-dimensional (2D) nanosheet morphology [15, 23, 24]. However, the details of the transformation from Ti-based MXene to Ti-based oxide and the cause of MXene’s instability in the water have been unclear. 2D materials were often employed to carry nanomaterials due to the large specific surface and fast ions/electrons diffusion [25–27]. Moreover, modification with carbon-based materials has been shown to be an effective solution for enhancing electron transport to achieve a high rate anode material [28, 29]. Recently, introducing a capacitive contribution has demonstrated another effective approach for overcoming poor sodium ion diffusion [7, 15, 23]. Designing defective nanomaterials can extremely enhance the capacitive contribution, leading to impressive rate performance and cycling stability [30, 31].

The architectural design of electrodes plays an important role in the performance and processing cost of batteries. Usually, the electrode is fabricated by a tedious slurry-casting process [32]. In a toxic and costly *N*-methyl pyrrolidone (NMP), the mixture of electrode materials including a conductive additive (~ 10 wt%) and an insulating binder (~ 10 wt%) is cast onto a heavy metal current collector. Inactive components occupy 70–80 wt% of the whole electrode, which significantly reduces the energy density and increases the cost [8, 33]. Thus, freestanding and binder-free construction could eliminate the additional inactive materials to realize a high-efficiency electrode.

Herein, a freestanding, binder-free, and defective Ti_2_CT_*x*_–MXene-derived TiO_2_ complex reduced graphene oxides (M-TiO_2_@rGO) 3D foam electrode was designed and synthesized via a simple and nontoxic hydrothermal process. Ti_2_CT_*x*_–MXene was converted to M-TiO_2_ by consuming the –OH in the H_2_O and the F functional group self-doped into the M-TiO_2_, during that a large number of lattice defects and oxygen vacancies were generated in situ. The addition of graphene oxide promoted the above reaction and constructed a 3D M-TiO_2_@graphene composite with a highly conductive pathway and accelerated ion diffusion. Directly employed as the anode for a sodium ion half-cell, the prepared materials displayed a remarkable rate ability and stable cycling performance due to its large capacitive contribution. Significantly, this composite was a capable and universal anode for both sodium ion full cells and sodium ion capacitors with favorable cycling stability.

## Experimental Section

### Preparation of Ti_2_CT_*x*_ MXene Powder

Ti_2_CT_*x*_ MXene was obtained by a synthetic HF solution. Specifically, 3 g of LiF (Alfa Aesar, 98.5%) was dissolved in 30 mL HCl (12 M). Then 2 g of Ti_2_AlC (200 mesh, purchased from Forsman Scientific (Beijing) Co., Ltd.) powder was slowly added into the above mixture solution and kept at 40 °C for 36 h under stirring. After that, the solution was centrifuged several times until the PH of the supernatant was about 5–6. Particularly, the supernatant of the first centrifuge was khaki. Finally, Ti_2_CT_*x*_ cake resembling graphene oxide  was obtained by freeze-dried.

### Preparation of M-TiO_2_@rGO Foam, M-TiO_2_, Na_3_V_2_(PO_4_)_3_, and HPAC

M-TiO_2_@rGO foam was prepared through a simple hydrothermal reaction [15]. In the typical preparation, 120 mg Ti_2_CT_*x*_ was dissolved in 40 mL distilled water and ultrasound for 3 h, then 130 mg GO (prepared from a modified Hummers method [34]) was added in it and continued ultrasound for 2 h. After that, 4 mL NaHSO_3_ (0.5 mM) solution was added to the above solution as a reducing agent. Finally, the above solution was transferred to a polytetrafluoroethylene reactor and kept at 180 °C for 16 h. The obtained M-TiO_2_@rGO cylindrical gel was dialyzed for 6 h in a mixture of water and ethanol. Finally, the M-TiO_2_@rGO foam was obtained through a freeze-drying process.

M-TiO_2_ nanoparticles were prepared under the same conditions without the addition of GO.

Na_3_V_2_(PO_4_)_3_ cathode material was prepared by the previous report [5].

3D hierarchical porous activated carbon (HPAC) derived from coir was prepared by the previous report [35].

### Material Characterizations

The compositions of M-TiO_2_@rGO and M-TiO_2_ were characterized by X-ray diffractometer (XRD) with copper Kα radiation (*λ* = 1.5418 Å) and Raman spectroscopy (RENISHAW, REF 2000, 514.5 nm laser). The surface functional groups of M-TiO_2_@rGO were checked by XPS (Axis Ultra DLD, Kratos Analytical). The morphologies of the M-TiO_2_@rGO, M-TiO_2_, and Ti_2_CT_*x*_ were analyzed by a field-emission scanning electron microscope (SEM, JEOL, JSM7500F) and a transmission electron microscope (TEM, JEOL, JEM-2100 model). Nitrogen adsorption–desorption isotherms were tested on a Quantachrome NOVA 2000e sorption analyzer at 77 K with liquid nitrogen. The rGO content of samples was tested by thermogravimetry (TA-Instruments-Wutersllc, TGA 500). The oxygen vacancies were checked through the electron paramagnetic resonance (EPR) spectra, which were recorded on a Bruker EPR ELEXSYS 500 spectrometer.

### Fabrication and Electrochemical Measurements of Half Cells

The self-supporting M-TiO_2_@rGO electrode sheets were obtained after freeze-drying the sheets cut from M-TiO_2_@rGO gel (when it was wet). The coated M-TiO_2_@rGO, HPAC, or NVP working electrodes consisted of active materials, supper P, and polyvinylidene fluoride (PVDF) (80:10:10 wt%), which were dispersed in *N*-methyl-2-pyrrolidone (NMP) uniformly. The M-TiO_2_ electrode was 70:20:10 wt% to balance its carbon-free nature. The slurry was coated on the current collectors (Cu foils for M-TiO_2_, M-TiO_2_@rGO anodes, and Al foils for HPAC, NVP cathodes) and dried at 80 °C in vacuum for 24 h. And then, these coated electrodes were cut into a circular shape with diameters of 12 mm. The mass loading was 0.5–1 mg cm^−2^ for anodes and 1–2 mg cm^−2^ for cathodes. In an Ar-filled glove box, CR2023-type coin cells were assembled with the as-prepared electrodes as working electrodes, the metallic sodium as the counter electrode and reference electrode, the glass fibers (Whatman) as separators, and the 1.0 M NaClO_4_ in EC: DMC: EMC = 1:1:1 vol% with 5.0% FEC as the electrolyte. The constant current charge/discharge tests were measured at the NEWARE battery test system (CT-4008 model), and the cyclic voltammetry (CV) tests were performed at Bio-Logic VMP3 electrochemical workstation. All electrochemical measurements were tested at room temperature. The specific capacities and current densities of self-supporting M-TiO_2_@rGO electrodes were calculated based on the M-TiO_2_ mass (70 wt%), correspondingly, the coated M-TiO_2_@rGO electrodes were calculated based on the rGO and M-TiO_2_ mass (80 wt% in the total coating materials). Those in cathode electrodes (HPAC and NVP) were based on the active materials (80 wt% in the total coating materials). The voltage ranges of the M-TiO_2_, M-TiO_2_@rGO, HPAC, and NVP were 0.1–3.0, 0.1–3.0, 3.0–4.2, and 2.0–4.3 V, respectively.

### Fabrication and Electrochemical Measurements of Full Cells of SICs and SIBs

Before assembling the full batteries, the coated M-TiO_2_@rGO was activated for 5 cycles at 50 mA g^−1^ in half cells. In the SICs, HPAC as a cathode and the coated M-TiO_2_@rGO as an anode, the mass ratios were 1:1.0, 1:1.5, 1:2.0, 1:3.0 (anode: cathode), and the voltage range was 1.0–4.0 V. In the SIBs, NVP as the cathode and the coated M-TiO_2_@rGO as the anode, the mass ratio was optimized at 1:3 (anode: cathode), and the voltage range was 1.0–3.5 V. The separator, electrolyte, and assembly method were the same as those in half cells.

The specific capacities of the SIBs were calculated based on the mass of the coated M-TiO_2_@rGO.

The energy densities (*E*, Wh kg^−1^) and power densities (P, W kg^−1^) of the SICs were calculated by Eqs. (1) and (2):

1 $$E = \frac{{\mathop \smallint \nolimits_{{t_{1} }}^{{t_{2} }} IV{\text{d}}t}}{{ \left( {M_{1} + M_{2} } \right)* 3.6}}$$

2 $$P = \frac{E}{T}$$

In the formulas, *t*_1_ and *t*_2_ are the start and end time (s) of the discharge, respectively, *I* (A) is the current, *V* (V) is the voltage at a particular time, *M*_1_ and *M*_2_ are the mass of anode and cathode (g), respectively, and *T* (h) is the total time of the discharge.

## Results and Discussion

### Synthesis and Characterization of Defective MXene-Derived TiO_2_@Graphene Electrode

**Fig. 1:**
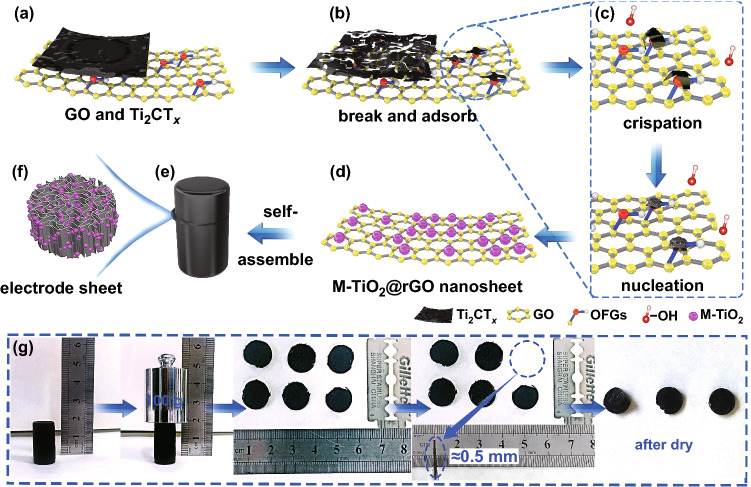
The schematic and digital pictures of the M-TiO_2_@rGO. **a** GO and Ti_2_CT_*x*_ nanosheet; **b** Ti_2_CT_*x*_ breaks and adsorbs on OFGs, and then **c** through crispation, finally, **d** reacts with OFGs and OH^−^ to form MXene-derived TiO_2_; **e** M-TiO_2_@rGO nanosheets through self-assemble process come into being composite gel; **f** internal structure of self-supporting electrode sheet; and **g** physical character of the M-TiO_2_@rGO gel and self-supporting electrodes

Ti_2_CT_*x*_–MXene-derived TiO_2_ complex graphene (M-TiO_2_@rGO) foam was prepared by a simple hydrothermal reaction associated with a series of transformations of Ti_2_CT_*x*_. First, Ti_2_CT_*x*_ nanosheets were mixed uniformly with graphene oxide (GO) nanosheets in an ultrasonic process (Fig. 1a). Then, in a hydrothermal process, the Ti_2_CT_*x*_ nanosheets broken into small lamellae and attached to the surface of GO by the inducing effect of the oxygen-containing function groups (OFGs) of the GO (Fig. 1b). As the reaction continued, the Ti_2_CT_*x*_ nanosheets gradually crimped, fractured (Fig. 1c), and finally converted into TiO_2_ nanoparticles (Fig. 1d). Compared to some preparation technologies using harmful organic solvents, this water-based synthetic approach was nontoxic and green. Meanwhile, GO was converted into reduced graphene oxides (rGO) and an M-TiO_2_@rGO gel was formed by a self-assembly process (Fig. 1e). As shown in the digital images (Fig. 1g), the obtained M-TiO_2_@rGO gel sample supported a 100 g weight, exhibiting good mechanical character. The gel also exhibited a certain toughness that could be cut into self-supporting electrodes with a thickness of ~ 0.5 mm. Furthermore, the electrode retained good mechanical properties after drying (Fig. S1). A schematic diagram of the internal structure of the electrode is shown in Fig. 1f, in which a perpendicular porous structure is favorable for electrolyte soaking and enhancing the contact area between electrode and electrolyte, leading to a capable electrochemical performance.

To understand the stability of Ti_2_CT_*x*_ and the formation mechanism of M-TiO_2_ in water, the product and the supernatant after an ultrasonic process were collected and examined. The pH of the supernatant was about 3–4, which suggested that much H^·^ was generated and OH^−^ was consumed (Fig. S2). Thus, the formation of M-TiO_2_ was mainly caused by the reactions between Ti_2_CT_*x*_ and OH^−^ produced by the water decomposition, which was accelerated by the applied energy (ultrasonic and thermal energy in this case). Such a reaction might explain the instability of Ti_2_CT_*x*_ in water and the unsatisfied electrocatalytic performance of Ti_2_CT_*x*_ in the hydrogen evolution reaction [36]. Meanwhile, the supernatant was slightly yellow, compared with distilled water (Fig. S2b), which might be caused by suspended carbon nanomaterials derived from Ti_2_CT_*x*_. Also, transmission electron microscopy (TEM) images of the Ti_2_CT_*x*_ products after different ultrasonic times showed pure Ti_2_CT_*x*_ with intact lamellar morphology (Fig. S3a). After 5 h of sonication, some pores were observed in Ti_2_CT_*x*_ nanosheets (Fig. S3b). With another 3 h of sonication, a large proportion of Ti_2_CT_*x*_ nanosheets cracked into nanoparticles, but a small portion remained in a lamellar state (Fig. S3c). Finally, Ti_2_CT_*x*_ nanosheets were completely converted into nanoparticles after 10 h of sonication (Fig. S3d) with 0.35 nm lattice spacing (Fig. S4), which was assigned to the (101) crystalline plane of anatase–TiO_2_, suggesting the formation of TiO_2_ nanoparticles [37]. In addition, the carbon layer could not be observed in the high rate TEM (HRTEM), further suggesting the carbon nanolayer exfoliation. Such a formation process was further confirmed by X-ray diffractometric (XRD) patterns of samples at different reaction times. First, Ti_2_CT_*x*_ displayed a (002) characteristic peak in place of the highest peak of the Ti_2_AlC at 39.5°, after the removal of Al layers in the Ti_2_AlC (Fig. S5) [38]. During the ultrasonic process, Ti_2_CT_*x*_ was converted into an intermediate product with an amorphous phase (after 5 h sonication) and finally formed the anatase M-TiO_2_ (Fig. S6). The microscopic appearance of M-TiO_2_ was observed by scanning electron microscopy (SEM), showing serious agglomeration (Fig. S7). Thus, 2D GO nanosheets were necessary because they not only provided much oxygen source and active sites for the formation of M-TiO_2_ but uniformly dispersed M-TiO_2_ to improve the electrochemical utilization rate.

**Fig. 2:**
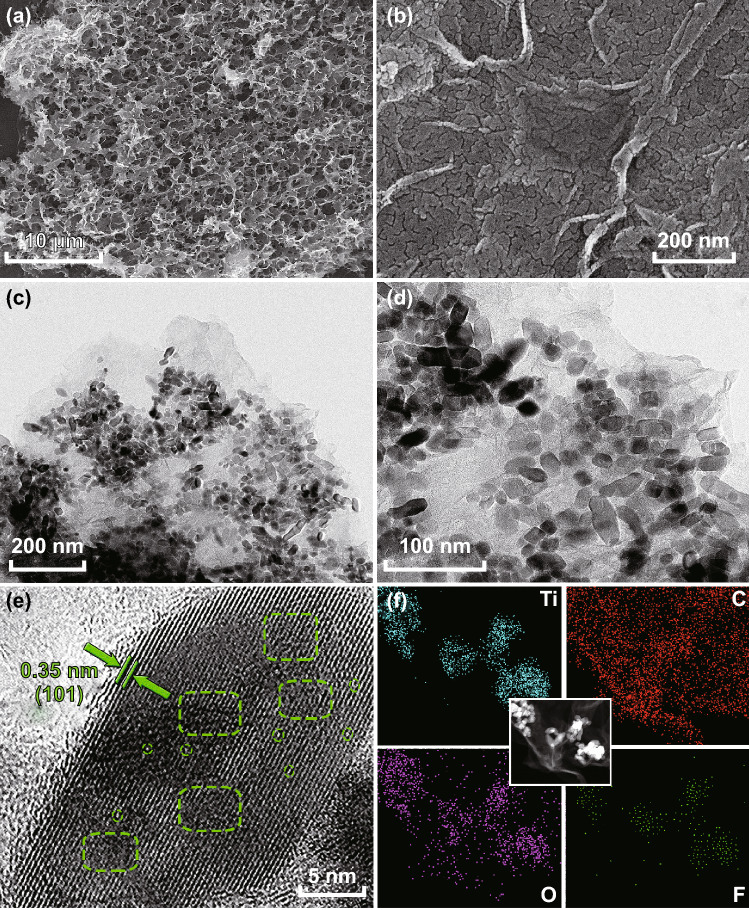
Microscopic morphology of M-TiO_2_@rGO foam. **a**, **b** SEM images; **c**, **d** TEM images; **e** HR-TEM image; and **f** dark field TEM and EDS mapping of C, O, Ti, and F

The morphology of the M-TiO_2_@rGO foam was observed by SEM and TEM, which presented a poriferous honeycomb-like structure composed of numerous M-TiO_2_/rGO nanosheets (Fig. 2a). On rGO nanosheets, M-TiO_2_ nanoparticles cracked from MXene was again confirmed and evenly distributed on the surface (Figs. 2b and S8). This structure of the horizontal arrangement would be beneficial for preserving the sheet structure of graphene and accelerating electronic and sodium ion transportation by the rGO base. TEM images of M-TiO_2_@rGO further confirmed the uniform distribution of TiO_2_ on the rGO nanosheets (Fig. 2c, d), with an average particle size of ~ 15 nm, which could shorten sodium ion transportation in the materials. The small difference in the particle size was attributed to the uneven dissociation of Ti_2_CT_*x*_ nanosheets. In addition, rGO had few-layers structure according to the wrinkle thickness and observable lattice fringes (Fig. S9). An HRTEM image displayed clear crystal planes with a *d*-spacing of 0.35 nm, corresponding to the (101) lattice plane of anatase–TiO_2_ [37]. Significantly, M-TiO_2_ presented a large number of lattice defects with distortion and vacancy (Figs. 2e and S10), which might fundamentally improve the ion/electronic conductivity of TiO_2_ [31]. Recent studies have also proved that defective engineering was highly beneficial to increasing reactive sites and improving ions diffusion, thereby enhancing electrochemical performance [19, 39]. To further investigate M-TiO_2_@rGO, EDS was performed, which showed the distribution of C, O, and Ti atoms in the composite. Interestingly, F inherited from Ti_2_CT_*x*_ surface groups was also observed in composite (Fig. 2f) and pure M-TiO_2_ (Fig. S11), which might be a cause for the defects in the TiO_2_. Moreover, pure anatase M-TiO_2_ exhibited a tan color rather than the white of commercial TiO_2_ (Fig. S12), and interestingly, peacock-blue rutile-TiO_2_ could be also synthesized by adjusting the pH and controlling the reaction conditions (Fig. S13), which was caused by the oxygen vacancy and heteroatom doping [19, 31, 40, 41].

**Fig. 3:**
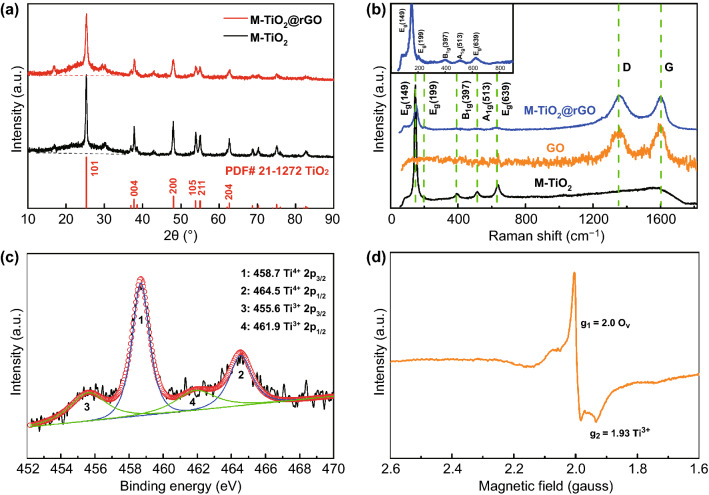
Structure characterization of M-TiO_2_@rGO composite. **a** XRD of M-TiO_2_ and M-TiO_2_@rGO; **b** Raman of M-TiO_2_, GO, and M-TiO_2_@rGO; **c** XPS curve of Ti *2p*; and **d** EPR curve of M-TiO_2_@rGO

The structures of M-TiO_2_ and M-TiO_2_@rGO foam were investigated by XRD (Fig. 3a). Peaks located at 25.3°, 37.8°, 48.0°, 53.9°, 55.0°, and 62.7° corresponded to (101), (004), (200), (105), (211), and (204) crystal face of anatase TiO_2_ (PDF#21-1272), respectively [7, 42]. The hump of M-TiO_2_@rGO at ~ 26° was enhanced due to the existence of rGO [15]. Using the Scherrer formula based on the XRD results [9], the average crystalline sizes of M-TiO_2_ and M-TiO_2_@rGO were calculated to be ~ 18 and 12 nm, respectively (Table S1). This implied that the addition of rGO limited the TiO_2_ growth. To further confirm the structure of M-TiO_2_@rGO, a Raman spectrum was performed (Fig. 3b), in which peaks located at 149, 199, 397, 513, and 639 cm^−1^ were assigned to the *E*_g_, *E*_g_, *B*_1g_, *A*_1g_, and *E*_g_ modes of anatase–TiO_2_, respectively [15], and the peaks at 1354 and 1600 cm^−1^ were denoted as the disorder and graphitic peaks (D and G bands, respectively) [37]. After the hydrothermal reaction, the *I*_D_/*I*_G_ of the M-TiO_2_@rGO composite (1.01) was higher than that of GO (0.92), which was assigned to the intercalation of M-TiO_2_ and nonstacking nature of M-TiO_2_@rGO compared to pure graphene [15, 43]. Meanwhile, the content of M-TiO_2_ in the composite was found to be ~ 67 wt%, according to thermogravimetric analysis (TG, Fig. S14). To understand the defect state and F doping, the X-ray photoelectron spectroscopy (XPS) test was performed (Figs. 3c and S15). From Fig. S15b, c, the attachment relation of M-TiO_2_ and rGO was confirmed by the presence of the Ti–O–C bond [44, 45]. This also indicated that the OFGs on GO was involved in the formation of M-TiO_2_. In addition, the F-Ti bond was observed at 684.6 eV due to F doping (Fig. S15a) [46]. Significantly, the Ti 2*p*_3/2_ and Ti 2*p*_1/2_ core level peaks could be divided into Ti^3+^ and Ti^4+^, due to lattice defects [37, 45]. In the oxygen signal, 530.5 eV could be assigned to lattice oxygen, and the obvious signal at 532.5 eV was caused by oxygen vacancies (Fig. S15c) [21]. Moreover, electron paramagnetic resonance (EPR) detection, a method to directly reveal oxygen vacancies, further confirmed the defect state (Fig. 3d). The strong signal at *g *= 2.0 confirmed the existence of abundant oxygen vacancies and *g *= 1.93 represented Ti^3+^ due to charge compensation during defective M-TiO_2_ formation [21, 37, 40]. These above results strongly revealed that the massive lattice defects were mainly caused by the presence of a large number of oxygen vacancies and F doping, which could be attributed to the advantages of using the MXene parent phase. Furthermore, M-TiO_2_@rGO presented a specific surface area of 174 m^2^ g^−1^ with an abundance of micropores and mesopores, shown by the nitrogen adsorption–desorption isotherms (Fig. S16). Such a hierarchical porous structure was very beneficial for electrolyte soaking, resulting in large contact areas between the electrode and electrolyte.

### Electrochemical Performance and Kinetics Analysis of M-TiO_2_@rGO Electrode

**Fig. 4:**
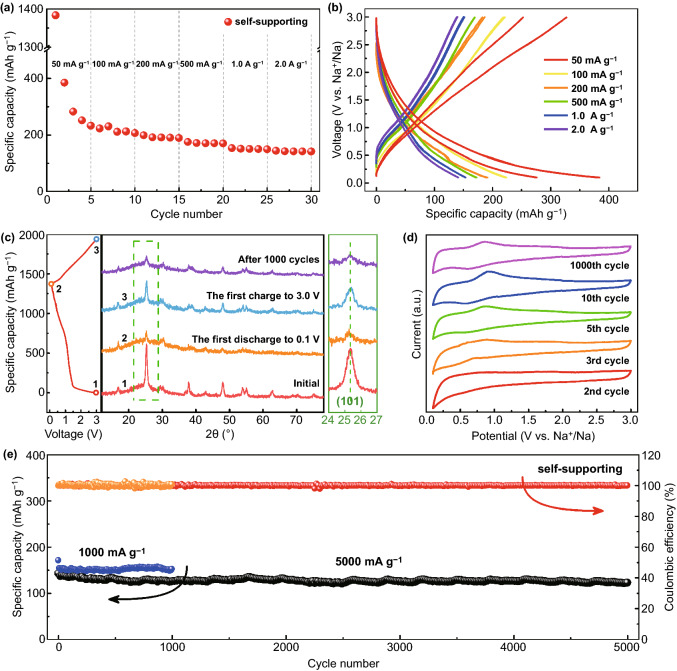
Electrochemical characterization of the self-supporting M-TiO_2_@rGO electrode in half cells. **a** Rate performance; **b** charge–discharge curves between 0.1 and 3.0 V from 50 mA g^−1^ to 2.0 A g^−1^; **c** XRD curves at different charging and discharging stages; **d** CV curves after galvanostatic charging-discharging tests with different cycles; and **e** 1000 and 5000 cycles cycling performance at 1.0 and 5.0 A g^−1^

M-TiO_2_@rGO foam could be applied directly as a self-supporting electrode. In sodium ion half cells, a remarkable rate performance was observed with average discharge specific capacities of 308, 223, 193, 171, 150, and 142 mAh g^−1^ at current densities of 50, 100, 200, 500, 1000, and 2000 mA g^−1^, respectively (Fig. 4a). The average voltage of the M-TiO_2_@rGO electrode was ~ 0.7 V (Fig. 4b), which effectively avoided dendrite generation. The sloping discharge profiles demonstrated a possible pseudocapacitive behavior [15]. In addition, a common irreversible capacity was observed in the first discharge and cyclic voltammetry (CV) curves (Fig. S17a, b), with a Coulombic efficiency of 28%. The irreversible capacity was related to some irreversible transformation between Ti^3+^ and Ti^4+^, irreversible insertion of sodium into porous structure, irreversible decomposition of the electrolyte, and the formation of solid electrolyte interphase (SEI) [15, 47, 48]. The *ex*-situ XRD curves of M-TiO_2_@rGO after the first discharge, first charge, and 1000 cycles showed that the peaks of M-TiO_2_@rGO were preserved, which demonstrated that the original structure remained unchanged during the sodiation or desodiation process (Fig. 4c). Even after 1000 cycles, the main peaks remained, suggesting favorable structural stability after repeated sodium ion insertion and desertion. Interestingly and typically, the M-TiO_2_@rGO electrode presented an electrochemical activation process during the initial cycles (Fig. 4d), in which the 2nd, 3rd, 5th, and 10th cycles represented after 2, 3, 5, and 10 cycles of galvanostatic charge–discharge testing (current density of 50 mA g^−1^), respectively. With cycling, a couple of anode/cathode peaks gradually appeared and became stable. Even after 1000 cycles, the anode/cathode peaks still remained well, suggesting a highly reversible sodium ion storage process. Such phenomena might have been caused by the transition of sodium ion storage from the surface to the bulk phase. This also suggested that M-TiO_2_@rGO presented multiple sodium ion storage mechanisms.

The cycling performance of the self-supporting M-TiO_2_@rGO electrode presented capacity retention of 97.2% after 1000 cycles at 1.0 A g^−1^ (Fig. 4e). Moreover, it demonstrated a capacity of 123.3 mAh g^−1^ with a capacity retention of 90.7% after 5000 cycles at 5.0 A g^−1^, corresponding to a decay of 0.018% per cycle, which was better than previous reports regarding sodium-based self-supporting anodes [8, 49–51]. From SEM images after 1000 cycles, M-TiO_2_ nanoparticles did not show shedding or agglomeration but only smooth edges, demonstrating good stability (Fig. S18). Electrochemical impedance spectroscopy (EIS) presented a reduced electrochemical reaction resistance and more pseudocapacitance diffusion behaviors after 1000 cycles (Fig. S19), which were attributed to activation process during the cycling. In addition, it was noticed that the capacity contribution from rGO was negligible (Fig. S20) and pure M-TiO_2_ exhibited a poor electrochemical performance due to severe agglomeration phenomenon (Fig. S21). Thus, the observed superior electrochemical performance of M-TiO_2_@rGO was attributed to the synergistic effects of rGO and defect-rich M-TiO_2_. The former provided a stable framework and enhanced electronic conductivity, while the later enhanced electrolyte soaking and provided many reaction active sites.

**Fig. 5:**
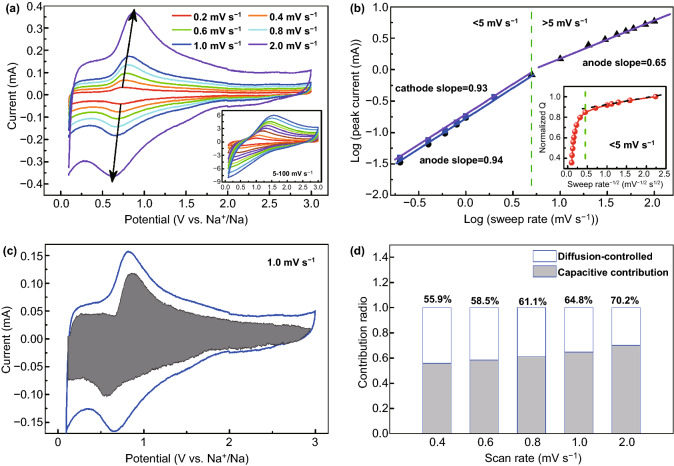
Kinetics analysis of sodium ion storage behavior in the M-TiO_2_@rGO electrode. **a** CV curves at different scan rates, inset: from 5 to 100 mV s^−1^; **b** *b*-value analysis through the relation of the peak current (*i*) and the scan rate (*v*); inset: normalized charge versus scan rate^−1/2^; **c** capacitive contribution area (gray) at the 1.0 mV s^−1^; and **d** capacitive contribution ratios at 0.4–2 mV s^−1^

To reveal the Na ion storage mechanism, a series of kinetic analyses for M-TiO_2_@rGO electrodes were performed as shown in Fig. 5. Figure 5a shows the CV curves from 0.2 to 100 mV s^−1^. The electrochemical kinetics of Na ion storage was analyzed through the relation of the peak current (*i*) and scan rate (*v*):

3 $$i = av^{b}$$

In Eq. (3), the *b*-value is between 0.5 and 1, in which the two ends represent the diffusion-controlled process and the capacitance-controlled process, respectively [15]. The *b*-value evaluated as the logarithm of Eq. (3) showed that the *b* of anode peaks was 0.94 and the *b* of the cathode was 0.93, at sweep rates range from 0.2 to 5.0 mV s^−1^ (Fig. 5b). This indicated that a capacitive process dominated the Na ion storage, leading to fast kinetics during charging and discharging. When the sweep rate was > 5.0 mV s^−1^, the *b* of the anode decreased to 0.65, implying that the diffusion process was the restrictive step [52]. The charge (*Q*) versus *v*^−1/2^ plot more intuitively illustrated the results (Fig. 5b, inset). When the sweep rate was < 5.0 mV s^−1^, the total charge did not change considerably with increased scan rates, which was because the capacitor behavior was less affected by the change of sweep speed. However, when the sweep rate was > 5 mV s^−1^, the charge decreased linearly with *v*^−1/2^, indicating that it was controlled by the diffusion process [22]. The above results suggested that the M-TiO_2_@rGO electrode contained two different types of sodium ion storage mechanisms and their presence directly affected the electrochemical performance. The ratios of the capacitance and diffusion contributions were determined using Eq. (4) [9]:

4 $$I\;({\text{V}}) = k_{1} v + k_{2} v^{1/2}$$

in which, *I*-value is the currents at a particular voltage and different scan rates, *v* is the scan rates, *k*_1_*v* and *k*_2_*v*^1/2^ represent surface and diffusion control, respectively [15]. The specific algorithms can be seen in the Supporting Information and the results showed in Figs. S22 and 5c, d. The CV area (gray) of capacitive contribution at 1.0 mV s^−1^ was displayed in Fig. 5c. The ratios of capacitive capacity showed a gradually increasing phenomenon as scan rates and a large capacitance contribution of 70.2% was obtained at 2.0 mV s^−1^ (Fig. 5d). Based on the above results, the observed capable rate and stable cycling performance could be ascribed to the improved electron conductivity and ions diffusion as well as a large pseudocapacitance contribution, arising from the in nanoscale and defect engineering.

### Sodium Ion Battery and Capacitor Based on M-TiO_2_@rGO Universal Anode

The M-TiO_2_@rGO foam electrode presented a lower working voltage, remarkable rate ability, and excellent cycling stability, demonstrating potential application as a universal anode for both SIBs and SICs. Thus, M-TiO_2_@rGO//Na_3_V_2_(PO_4_)_3_ SIBs and M-TiO_2_@rGO//biomass-derived active carbon SICs were designed and assembled.

A SIB with Na_3_V_2_(PO_4_)_3_ (NVP) as the cathode and activated M-TiO_2_@rGO as the anode was assembled. Before that, the electrochemical performance of NVP was investigated in a Na ion half-cell at the voltage window of 2.0–4.3 V. The charge/discharge profiles at different current densities were shown in Fig. S23a, with a charging platform of 3.4 V and a discharging platform of 3.3 V at 50 mA g^−1^. A stable cycling performance with capacity retention of 85% was obtained after 200 cycles at 500 mA g^−1^ (Fig. S23b). To better match the qualities of anode and cathode to ensure electrochemical performance, M-TiO_2_@rGO was coated onto copper foil to flexibly regulate the mass loads of active materials. The rate performance of the coating-M-TiO_2_@rGO electrode showed higher specific capacities than self-supporting M-TiO_2_@rGO electrodes and other published reports at the same conditions (Fig. S24a). Furthermore, this half-cell exhibited an amazing cycling performance. At an ultra-high current density of 10 A g^−1^, a capacity of 127.2 mAh g^−1^ and capacity retention of 84.6% were still obtained after 10,000 cycles (Fig. S24b).

**Fig. 6:**
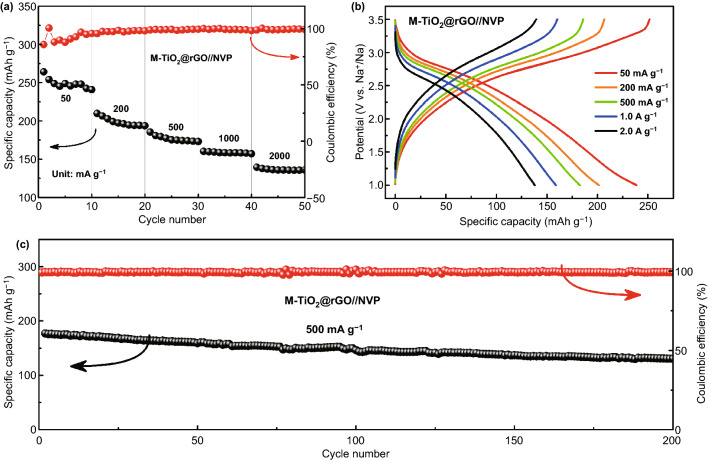
Electrochemical performance of the sodium ion batteries (SIBs) with the NVP as cathodes and coated M-TiO_2_@rGO as anodes. **a** Rate performance; **b** charge–discharge curves at different current densities; and **c** cycling performance at 500 mA∙g^−1^

The electrochemical performance of SIBs was optimized by setting the electrode mass ratio at 1:3 (anode: cathode). The galvanostatic charge–discharge test was operated at 1.0–3.4 V. The M-TiO_2_@rGO//NVP full cell presented average capacities of 247.4, 200.3, 178.3, 148.3, and 135.8 mAh g^−1^ at current densities of 50, 200, 500, 1000, and 2000 mA g^−1^, respectively, demonstrating a good rate performance (Fig. 6a). With current density increased, the shapes of the charge/discharge profiles retained well (Fig. 6b). In addition, the cycle stability was tested at the current density of 500 mA g^−1^ (Fig. 6c). The TiO_2_@rGO//NVP full cell exhibited a discharge capacity of 177.1 mAh g^−1^ in the first cycle and capacity retention of 74% (130.7 mAh g^−1^) after 200 cycles. Meanwhile, the Coulomb efficiency tended to 100%, indicating a perfect kinetics match between the cathode and anode. It should be noted that the decreased capacity of the full cell was mainly caused by the cathode, based on the results of half cells. Thus, if M-TiO_2_@rGO was matched with other more stable cathodes, the electrochemical performance would be further improved.

**Fig. 7:**
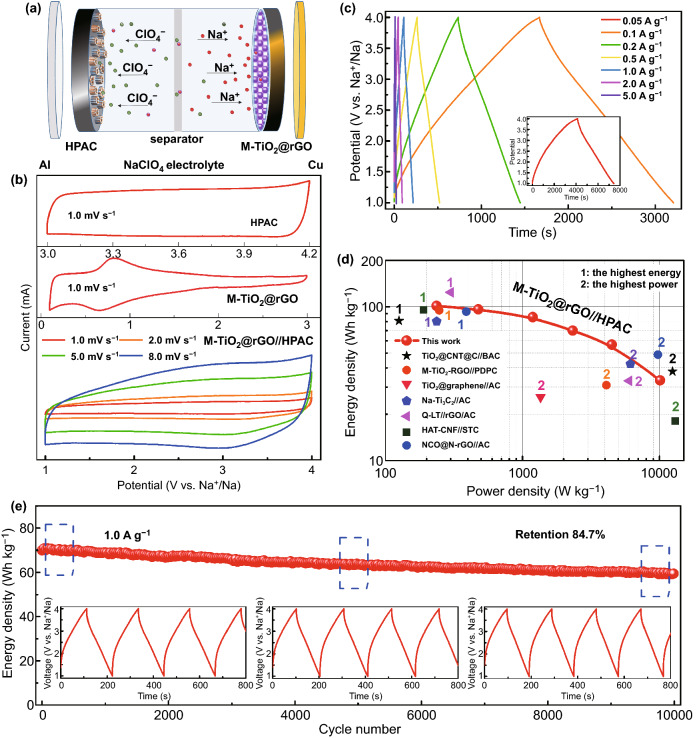
Electrochemical performance of the sodium ion capacitors (SICs) with HPAC as cathodes and coated M-TiO_2_@rGO as anodes. **a** Schematic of the SICs; **b** CV curves of HPAC, M-TiO_2_@rGO, and M-TiO_2_@rGO//HPAC; **c** CP curves at different current densities; **d** Ragone plot of power density and energy density; and **e** cycling performance at 1.0 A g^−1^, inset: CP curves at different states

To achieve Na-based energy storage devices with higher power density, a SIC was assembled with activated M-TiO_2_@rGO as the anode and a 3D hierarchical porous activated carbon (HPAC) derived from coir [35] as the cathode. The electrochemical performance of the HPAC in Na-ion half-cell was measured at 3.0–4.2 V, with outstanding rate and cycling performances (Fig. S25). A typical electric double layer capacitor (EDLC) behavior of HPAC was demonstrated in the rectangular CV curves and triangular chronopotential potential (CP) curves (Fig. S25a, c). To better match the quality between the positive and negative electrodes to ensure electrochemical performance, the coating-M-TiO_2_@rGO electrode was used as the anode. The schematic of the SIC is shown in Fig. 7a. In the charging stage, Na^+^ intercalated into the M-TiO_2_@rGO anode from the electrolyte, while ClO_4_^−^ anions absorbed on the HPAC cathode to balance the charge in electrolyte [53]. During the discharge stage, a reverse reaction occurred. To obtain higher energy density and cycling stability, the working voltage range of the SIC was selected to be 1.0–4.0 V [23]. The optimal mass ratio was investigated via rate and cycling performance tests (Fig. S26), which was set at 1:1.0, 1:1.5, 1:2.0, and 1:3.0 for anode/cathode. The device with a mass ratio of 1:1.5 displayed higher energy density and the best cycling performance, with capacity retention of 88.6% after 1000 cycles (2.0 A g^−1^).

The optimized SICs with a mass ratio of 1:1.5 and an operating voltage window of 1.0–4.0 V were chosen to further estimate the electrochemical performance (Fig. 7). The CV curves before and after assembly are shown in Fig. 7b, and the CV curves of SIC displayed an approximate rectangle with a slight peak attributed to certain pseudocapacitance reactions at high voltage, which was beneficial for achieving high energy density. Based on galvanostatic charge–discharge measurements, the typical near-linear CP curves at 1.0–4.0 V indicated a near ideal capacitive property, which implied rapid ion storage kinetics (Fig. 7c) [56]. The energy and power densities were calculated based on the total masses of the anode and cathode. A superhigh energy density of 124.3 Wh kg^−1^ was obtained in the first cycle. After 5 cycles activation process at 0.05 A g^−1^, a high energy density of 101.2 Wh kg^−1^ was achieved at a power density of 236.0 W kg^−1^ (0.1 A g^−1^) and an ultrahigh power density of 10,103.7 W kg^−1^ was obtained at an energy density of 33.2 Wh kg^−1^ (5.0 A g^−1^). Such a remarkable electrochemical performance was at the top level compared to previous SICs reports (Fig. 7d), such as TiO_2_@CNT@C//BAC [9], M-TiO_2_–RGO//PDPC [23], TiO_2_@graphene//AC [7], HAT-CNF//STC [54], Na-Ti_3_C_2_//AC [55], NCO@N-rGO//AC [47], and Q-LT//rGO/AC [53]. Moreover, these SICs presented a long-term stable cycling performance with an energy retention ratio of 84.7% after 10,000 cycles at 1.0 A g^−1^ (Fig. 7e). In addition, no deformation occurred in the charge/discharge profiles, which further illustrated an excellent reversibility and perfect dynamics match between the anode and cathode. The above results suggest favorable designs are found here for the anode and cathode of Na-ion capacitors, which will provide a feasible strategy for the practical application of sodium ion energy storage devices.

## Conclusions

In summary, Ti_2_CT_*x*_-derived defective TiO_2_ nanoparticles were synthesized via a facile and environmentally friendly approach, Ti_2_CT_*x*_ MXene aqueous phase splitting, in which the TiO_2_ formation mechanism of Ti_2_CT_*x*_ binding to OH^−^ produced by water decomposition was first revealed. Employing MXene parent phase achieved rich oxygen vacancies and F-doped metal oxides nanoparticles in situ, which fundamentally improved electrons/ions conductivity. Furthermore, an M-TiO_2_@rGO foam was synthesized and exhibited a hierarchical porous structure, which benefitted electrolyte permeation and enhanced ion transport. Employed as a free-standing anode for Na ion half cells, this material showed excellent rate performance and ultra-long cycling stability, with a retention ratio of 90.7% even after 5000 cycles at 5.0 A g^−1^. Electrochemical analysis demonstrated pseudocapacitance-dominated hybrid sodium storage mechanism. At 2.0 mV s^−1^, capacitive contribution is ~ 70%, which was caused by the defects and nano-size particles. Finally, the distinctive M-TiO_2_@rGO electrode displayed a suitable electrochemical performance as a universal anode for both M-TiO_2_@rGO//NVP SIBs and M-TiO_2_@rGO//HPAC SICs. The SICs showed a high energy density of 101.2 Wh kg^−1^ at a power density of 236.0 W kg^−1^ and an ultrahigh power density of 10,103.7 W kg^−1^ at a mezzo energy density of 33.2 Wh kg^−1^, along with outstanding cyclic stability with energy retention ratio of 84.7% after 10,000 cycles at 1.0 A g^−1^. This study will facilitate the rapid development of anodes for high-performance sodium-based energy storage devices and inspire more applications for MXene.

## Electronic supplementary material

Below is the link to the electronic supplementary material.

Supplementary material 1 (PDF 2065 kb)

## References

[1] Dunn B, Kamath H, Tarascon J-M (2011). Electrical energy storage for the grid: a battery of choices. Science.

[2] Park M, Ryu J, Wang W, Cho J (2017). Material design and engineering of next-generation flow-battery technologies. Nat. Rev. Mater..

[3] Vaalma C, Buchholz D, Weil M, Passerini S (2018). A cost and resource analysis of sodium-ion batteries. Nat. Rev. Mater..

[4] Li S, Zhao Z, Li C, Liu Z, Li D (2019). SnS_2_@C hollow nanospheres with robust structural stability as high-performance anodes for sodium ion batteries. Nano-Micro Lett..

[5] Jiang Y, Zhou X, Li D, Cheng X, Liu F, Yu Y (2018). Highly reversible Na storage in Na_3_V_2_(PO4)_3_ by optimizing nanostructure and rational surface engineering. Adv. Energy Mater..

[6] Zhao J, Yang X, Yao Y, Gao Y, Sui Y (2018). Moving to aqueous binder: a valid approach to achieving high-rate capability and long-term durability for sodium-ion battery. Adv. Sci..

[7] Le Z, Liu F, Nie P, Li X, Liu X (2017). Pseudocapacitive sodium storage in mesoporous single-crystal-like TiO_2_–graphene nanocomposite enables high-performance sodium-ion capacitors. ACS Nano.

[8] Xia Z, Sun H, He X, Sun Z, Lu C (2019). In situ construction of CoSe_2_@vertical-oriented graphene arrays as self-supporting electrodes for sodium-ion capacitors and electrocatalytic oxygen evolution. Nano Energy.

[9] Zhu YE, Yang L, Sheng J, Chen Y, Gu H, Wei J, Zhou Z (2017). Fast sodium storage in TiO_2_@CNT@C nanorods for high-performance Na-ion capacitors. Adv. Energy Mater..

[10] Sun Y, Guo S, Zhou H (2018). Exploration of advanced electrode materials for rechargeable sodium-ion batteries. Adv. Energy Mater..

[11] Wu F, Zhao C, Chen S, Lu Y, Hou Y, Hu YS, Maier J, Yan Y (2018). Multi-electron reaction materials for sodium-based batteries. Mater. Today.

[12] Peng C, Chen H, Zhong G, Tang W, Xiang Y (2019). Capacity fading induced by phase conversion hysteresis within alloying phosphorus anode. Nano Energy.

[13] Bai P, He Y, Xiong P, Zhao X, Xu K, Xu Y (2018). Long cycle life and high rate sodium-ion chemistry for hard carbon anodes. Energy Storage Mater..

[14] Guo S, Li Q, Liu P, Chen M, Zhou H (2017). Environmentally stable interface of layered oxide cathodes for sodium-ion batteries. Nat. Commun..

[15] Fang Y, Hu R, Liu B, Zhang Y, Zhu K (2019). Mxene-derived TiO_2_/reduced graphene oxide composite with an enhanced capacitive capacity for Li-ion and K-ion batteries. J. Mater. Chem. A.

[16] Sang L, Lei L, Burda C (2019). Electrochemical fabrication of rGO-embedded Ag–TiO_2_ nanoring/nanotube arrays for plasmonic solar water splitting. Nano-Micro Lett..

[17] Gan Q, He H, Zhu Y, Wang Z, Qin N, Gu S, Li Z, Luo W, Lu Z (2019). Defect-assisted selective surface phosphorus doping to enhance rate capability of titanium dioxide for sodium ion batteries. ACS Nano.

[18] Li C, Wang T, Zhao ZJ, Yang W, Li JF (2018). Promoted fixation of molecular nitrogen with surface oxygen vacancies on plasmon-enhanced TiO_2_ photoelectrodes. Angew. Chem. Int. Ed..

[19] Zhang Y, Ding Z, Foster CW, Banks CE, Qiu X, Ji X (2017). Oxygen vacancies evoked blue TiO_2_ (B) nanobelts with efficiency enhancement in sodium storage behaviors. Adv. Funct. Mater..

[20] Huang L, Zhou X, Xue R, Xu P, Wang S (2020). Low-temperature growing anatase TiO_2_/SnO_2_ multi-dimensional heterojunctions at MXene conductive network for high-efficient perovskite solar cells. Nano-Micro Lett..

[21] Zhang W, Cai L, Cao S, Qiao L, Zeng Y (2019). Electrode materials: interfacial lattice-strain-driven generation of oxygen vacancies in an aerobic-annealed TiO_2_ (B) electrode. Adv. Mater..

[22] Li B, Xi B, Feng Z, Lin Y, Liu J, Feng J, Qian Y, Xiong S (2018). Hierarchical porous nanosheets constructed by graphene-coated, interconnected TiO_2_ nanoparticles for ultrafast sodium storage. Adv. Mater..

[23] Wang R, Wang S, Zhang Y, Jin D, Tao X, Zhang L (2018). Graphene-coupled Ti_3_C_2_ MXenes-derived TiO_2_ mesostructure: promising sodium-ion capacitor anode with fast ion storage and long-term cycling. J. Mater. Chem. A.

[24] Zhang X, Li J, Li J, Han L, Lu T, Zhang X, Zhu G, Pan L (2020). 3D TiO_2_@nitrogen-doped carbon/Fe_7_S_8_ composite derived from polypyrrole-encapsulated alkalized MXene as anode material for high-performance lithium-ion batteries. Chem. Eng. J..

[25] Nakhanivej P, Yu X, Park SK, Kim S, Hong J-Y (2019). Revealing molecular-level surface redox sites of controllably oxidized black phosphorus nanosheets. Nat. Mater..

[26] Yu X, Yun S, Yeon JS, Bhattacharya P, Wang L, Lee SW, Hu X, Park HS (2018). Emergent pseudocapacitance of 2D nanomaterials. Adv. Energy Mater..

[27] Fang Y, Zhang Y, Zhu K, Lian R, Gao Y (2019). Lithiophilic three-dimensional porous Ti_3_C_2_T_*x*_–rGO membrane as a stable scaffold for safe alkali metal (Li or Na) anodes. ACS Nano.

[28] He H, Gan Q, Wang H, Xu G-L, Zhang X (2018). Structure-dependent performance of TiO_2_/C as anode material for Na-ion batteries. Nano Energy.

[29] Xing Y, Wang S, Fang B, Song G, Wilkinson DP, Zhang S (2018). N-doped hollow urchin-like anatase TiO_2_@C composite as a novel anode for Li-ion batteries. J. Power Sources.

[30] Zhu Y, Peng L, Fang Z, Yan C, Zhang X, Yu G (2018). Structural engineering of 2D nanomaterials for energy storage and catalysis. Adv. Mater..

[31] Naldoni A, Allieta M, Santangelo S, Marelli M, Fabbri F (2012). Effect of nature and location of defects on bandgap narrowing in black TiO_2_ nanoparticles. J. Am. Chem. Soc..

[32] Wu Q, Zheng JP, Hendrickson M, Plichta EJ (2019). Dry process for fabricating low cost and high performance electrode for energy storage devices. MRS Adv..

[33] Wang S, Wang Q, Zeng W, Wang M, Ruan L, Ma Y (2019). A new free-standing aqueous zinc-ion capacitor based on MnO_2_–CNTs cathode and MXene anode. Nano-Micro Lett..

[34] Zhu Y, Murali S, Cai W, Li X, Suk JW, Potts JR, Ruoff RS (2010). Graphene and graphene oxide: synthesis, properties, and applications. Adv. Mater..

[35] Yin L, Chen Y, Zhao X, Hou B, Cao B (2016). 3-Dimensional hierarchical porous activated carbon derived from coconut fibers with high-rate performance for symmetric supercapacitors. Mater. Design.

[36] Seh ZW, Fredrickson KD, Anasori B, Kibsgaard J, Strickler AL (2016). Two-dimensional molybdenum carbide (MXene) as an efficient electrocatalyst for hydrogen evolution. ACS Energy Lett..

[37] He H, Zhang Q, Wang H, Zhang H, Li J, Peng Z, Tang Y, Shao M (2017). Defect-rich TiO_2_-δ nanocrystals confined in a mooncake-shaped porous carbon matrix as an advanced Na ion battery anode. J. Power Sources.

[38] Xu H, Yin X, Li X, Li M, Liang S, Zhang L, Cheng L (2019). Lightweight Ti_2_CT_x_ MXene/poly (vinyl alcohol) composite foams for electromagnetic wave shielding with absorption dominated feature. ACS Appl. Mater. Interfaces..

[39] Zhao Y, Zhao Y, Shi R, Wang B, Waterhouse GI, Wu LZ, Tung CH, Zhang T (2019). Tuning oxygen vacancies in ultrathin TiO_2_ nanosheets to boost photocatalytic nitrogen fixation up to 700 nm. Adv. Mater..

[40] Yang Y, Ye K, Cao D, Gao P, Qiu M, Liu L, Yang P (2018). Efficient charge separation from F-selective etching and doping of anatase–TiO_2_ {001} for enhanced photocatalytic hydrogen production. ACS Appl. Mater. Interfaces..

[41] Hüttenhofer L, Eckmann F, Lauri A, Cambiasso J, Pensa E (2020). Anapole excitations in oxygen vacancy-rich TiO_2−*x*_ nanoresonators: tuning the absorption for photocatalysis in the visible. ACS Nano.

[42] Ren H, Yu R, Qi J, Zhang L, Jin Q, Wang D (2019). Hollow multishelled heterostructured anatase/TiO_2_ (B) with superior rate capability and cycling performance. Adv. Mater..

[43] Kumar NA, Gaddam RR, Varanasi SR, Yang D, Bhatia SK, Zhao X (2016). Sodium ion storage in reduced graphene oxide. Electrochim. Acta.

[44] Ahmed B, Anjum DH, Hedhili MN, Gogotsi Y, Alshareef HN (2016). H_2_O_2_ assisted room temperature oxidation of Ti_2_C MXene for Li-ion battery anodes. Nanoscale.

[45] Chen C, Wen Y, Hu X, Ji X, Yan M (2015). Na^+^ intercalation pseudocapacitance in graphene-coupled titanium oxide enabling ultra-fast sodium storage and long-term cycling. Nat. Commun..

[46] Khan JA, Han C, Shah NS, Khan HM, Nadagouda MN (2014). Ultraviolet–visible light–sensitive high surface area phosphorous–fluorine–co-doped TiO_2_ nanoparticles for the degradation of atrazine in water. Environ. Eng. Sci..

[47] Yang D, Zhao Q, Huang L, Xu B, Kumar NA, Zhao XS (2018). Encapsulation of NiCo_2_O_4_ in nitrogen-doped reduced graphene oxide for sodium ion capacitors. J. Mater. Chem. A.

[48] Yan D, Yu C, Li D, Zhang X, Li J, Lu T, Pan L (2016). Improved sodium-ion storage performance of TiO_2_ nanotubes by Ni^2+^ doping. J. Mater. Chem. A.

[49] Wang L, Yang G, Wang J, Wang S, Wang C, Peng S, Yan W, Ramakrishna S (2019). In situ fabrication of branched TiO_2_/C nanofibers as binder-free and free-standing anodes for high-performance sodium-ion batteries. Small.

[50] Xu G, Yang L, Li Z, Wei X, Chu PK (2017). Protein-assisted assembly of mesoporous nanocrystals and carbon nanotubes for self-supporting high-performance sodium electrodes. J. Mater. Chem. A.

[51] Xu G, Tian Y, Wei X, Yang L, Chu PK (2017). Free-standing electrodes composed of carbon-coated Li_4_Ti_5_O_12_ nanosheets and reduced graphene oxide for advanced sodium ion batteries. J. Power Sources.

[52] Augustyn V, Come J, Lowe MA, Kim JW, Taberna P-L, Tolbert SH, Abruña HD, Simon P, Dunn B (2013). High-rate electrochemical energy storage through Li^+^ intercalation pseudocapacitance. Nat. Mater..

[53] Que LF, Yu FD, Sui XL, Zhao L, Zhou JG, Gu D-M, Wang Z-B (2019). Thermal-induced interlayer defect engineering toward super high-performance sodium ion capacitors. Nano Energy.

[54] Yan R, Josef E, Huang H, Leus K, Niederberger M (2019). Understanding the charge storage mechanism to achieve high capacity and fast ion storage in sodium-ion capacitor anodes by using electrospun nitrogen-doped carbon fibers. Adv. Funct. Mater..

[55] Luo J, Cong F, Jin C, Yuan H, Sheng O (2018). Tunable pseudocapacitance storage of MXene by cation pillaring for high-performance sodium ion capacitors. J. Mater. Chem. A.

[56] Kumar NA, Baek J-B (2015). Doped graphene supercapacitors. Nanotechnology.

