# Supplementary material for: MXene-Derived Defect-Rich TiO2@rGO as High-Rate Anodes for Full Na Ion Batteries and Capacitors
Source: Nanomicro Lett. 2020 Jun 16;12:128. doi: 10.1007/s40820-020-00471-9 (PMC7770766; doi:10.1007/s40820-020-00471-9)
Supplement: Supplementary file 1 — Supplementary material 1 (PDF 2065 kb) [file 40820_2020_471_MOESM1_ESM.pdf]

Supporting Information for

MXene-Derived Defect-Rich TiO_2@rGO as High-Rate Anodes for Full Na-Ion Batteries and Capacitors

Yongzheng Fang[1], Yingying Zhang [1], Chenxu Miao [1], Kai Zhu [1, *], Yong Chen [2], Fei Du [3], Jinling Yin [1], Ke Ye [1], Kui Cheng [1], Jun Yan [1], Guiling Wang [1], Dianxue Cao [1, *]

[1]Key Laboratory of Superlight Materials and Surface Technology (Ministry of Education), College of Material Science and Chemical Engineering, Harbin Engineering University, Harbin 150001, People's Republic of China

[2]State Key Laboratory of Marine Resource Utilization in South China Sea, Hainan Provincial Key Laboratory of Research on Utilization of Si-Zr-Ti Resources, College of Materials Science and Engineering, Hainan University, 58 Renmin Road, Haikou, 570228, People's Republic of China

[3]Key Laboratory of Physics and Technology for Advanced Batteries (Ministry of Education), College of Physics, Jilin University, Changchun 130012, People's Republic of China.

*Corresponding authors. E-mail: kzhu@hrbeu.edu.cn (Kai Zhu); caodianxue@hrbeu.edu.cn (Dianxue Cao)

Supplementary Table and Figures

Table S1 Size determination of the TiO_2 in M-TiO_2@rGO and M-TiO_2

Sample	Index	2θ (degree)	β (degree)	Length(nm)
M-TiO_2	(101)	25.28	0.441	18.27
M-TiO_2@rGO	(101)	25.28	0.620	12.68

Calculation Method

Based on the XRD dates in Fig. 3a. Through the Scherrer equation:

$$\text{Diameter} = 0.89*\lambda/(\beta*\cos\theta) \qquad (1)$$

where λ is the wavelength of the X-ray (0.15418 nm) and β is the full width at half maximum of the diffraction peak (radian) [S1]. The average sizes were 12.68 nm and 18.27 nm for M-TiO_2@rGO and M-TiO_2, respectively

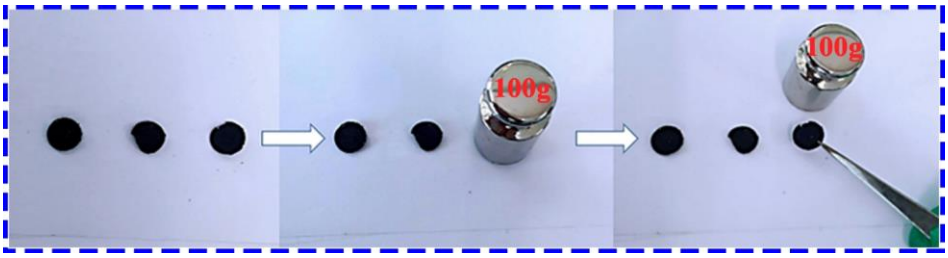

Fig. S1 Mechanical properties of the self-supporting M-TiO_2@rGO electrode sheets. After 100 g weight pressed, the electrode sheets were still intact

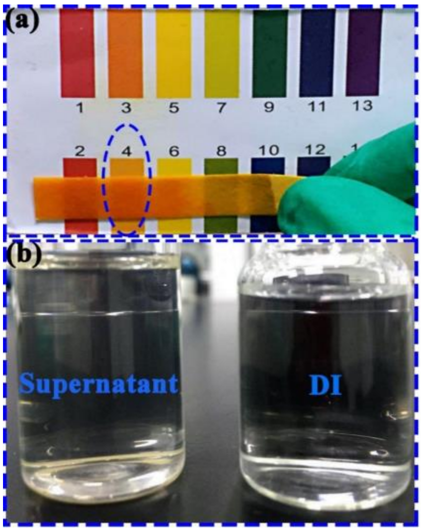

Fig. S2 Properties of the supernatant after M-TiO_2 formation

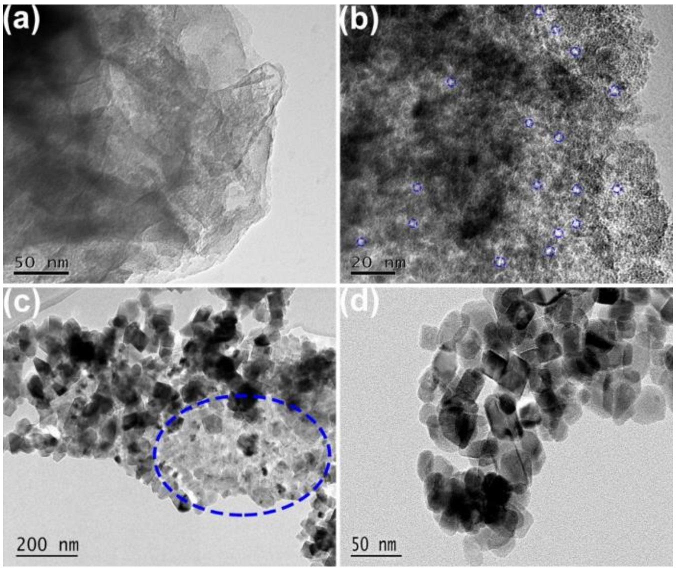

Fig. S3 TEM images of the change process from Ti_2CT_x to M-TiO_2. (**a**) The initial Ti_2CT_x image. (**b**) After sonication for 5 hours in distilled water, it could be seen that many nanopores on the Ti_2CT_x. (**c**) After sonication for 8 hours, most of the Ti_2CT_x nanosheet had converted into M-TiO_2 nanoparticles, but a small area remained lamellar. (**d**) After sonication for 10 h, M-TiO_2 nanoparticles formed completely

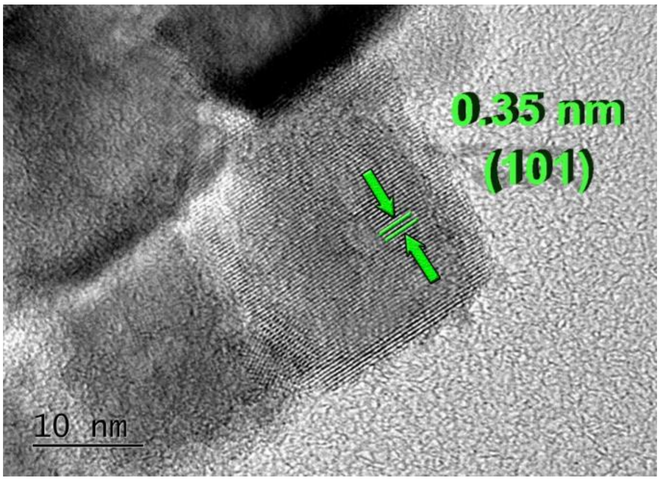

Fig. S4 HR-TEM image of the M-TiO_2

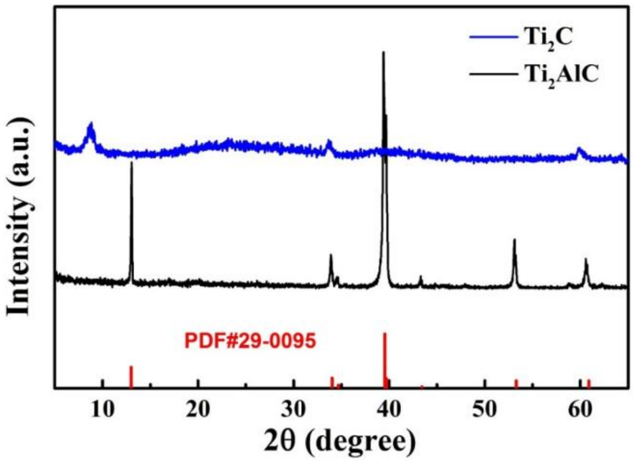

Fig. S5 XRD profiles of Ti_2AlC and Ti_2CT_x

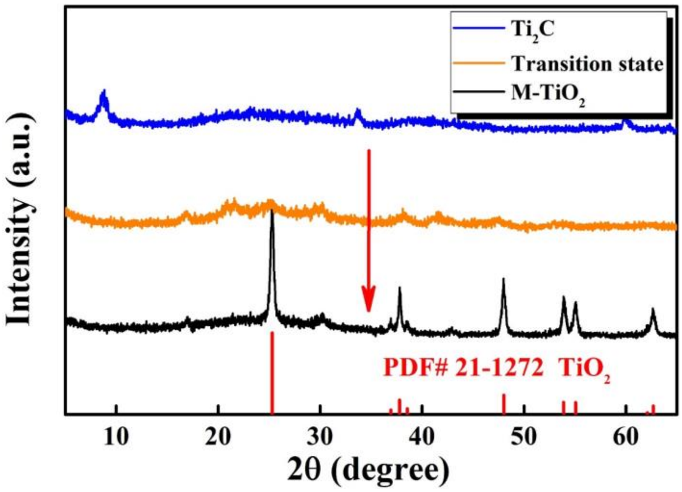

Fig. S6 XRD curves for the formation process of M-TiO_2. It shows the change processes from the Ti_2CT_x nanosheets (blue) to the amorphous phase (yellow) to the M-TiO_2 (black)

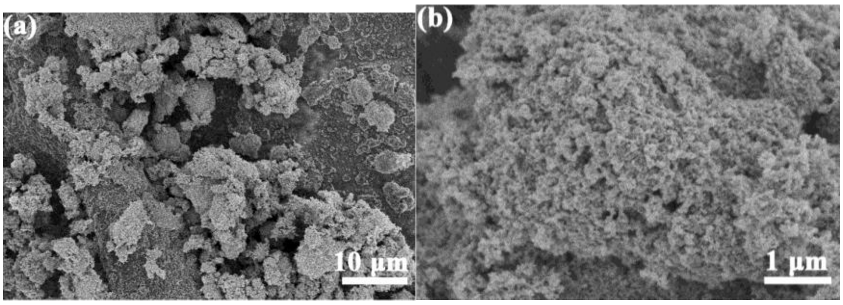

Fig. S7 SEM images of the M- TiO_2 with a serious agglomeration

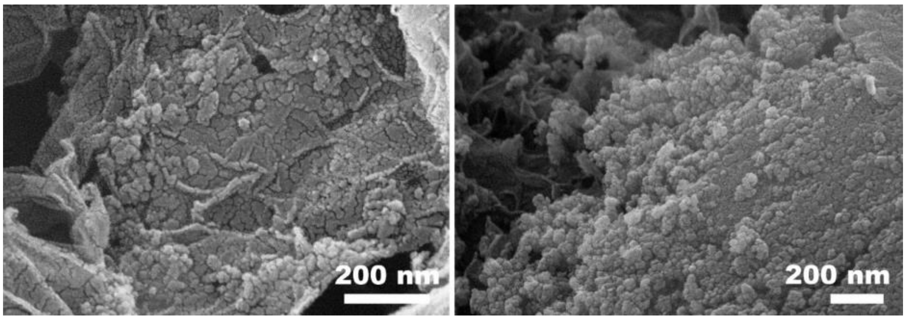

Fig. S8 Uniformly dispersed M-TiO_2 nanoparticles on rGO nanosheets

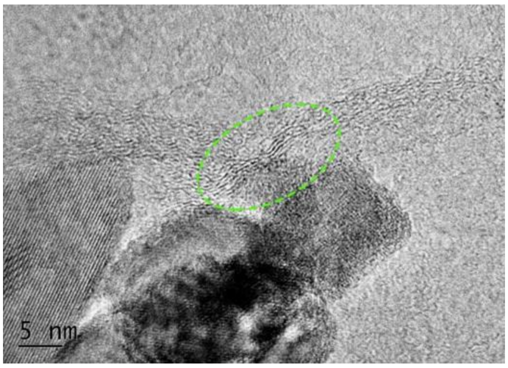

Fig. S9 HR-TEM image of lattice fringes of rGO in M-TiO_2@rGO

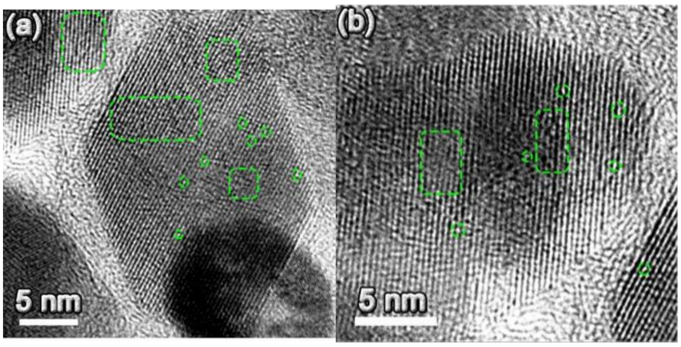

Fig. S10 HR-TEM images of lattice defects of TiO_2 in M-TiO_2@rGO. Lattice stripes exhibit a lot of distortion, blur, and breakage.

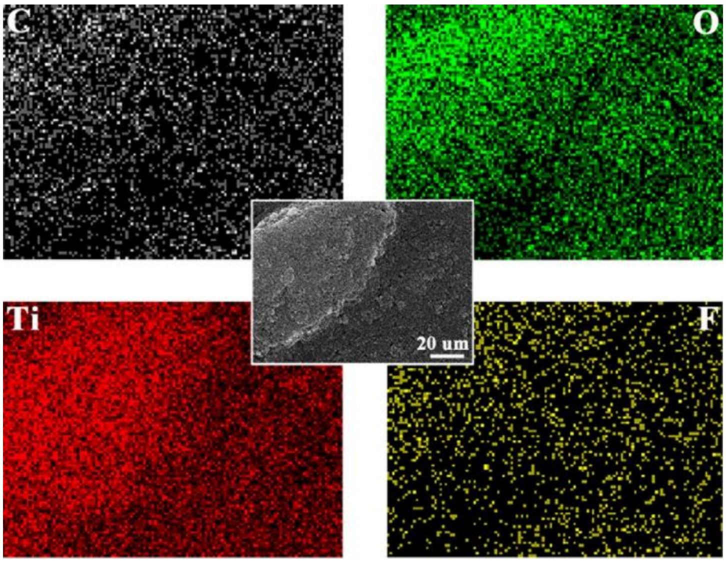

Fig. S11 EDS mapping images of SEM of pure M-TiO_2

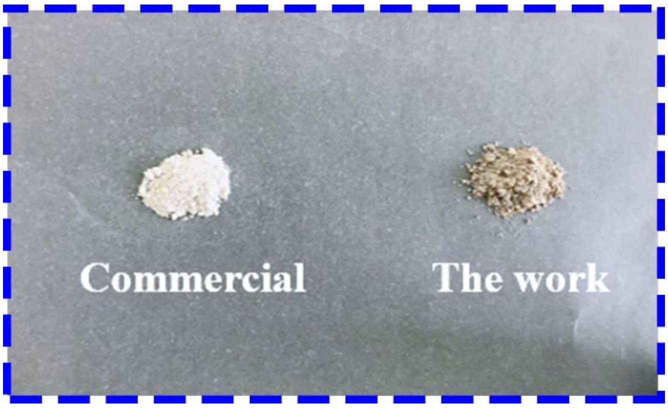

Fig. S12 The difference between commercial TiO_2 and M-TiO_2 in color

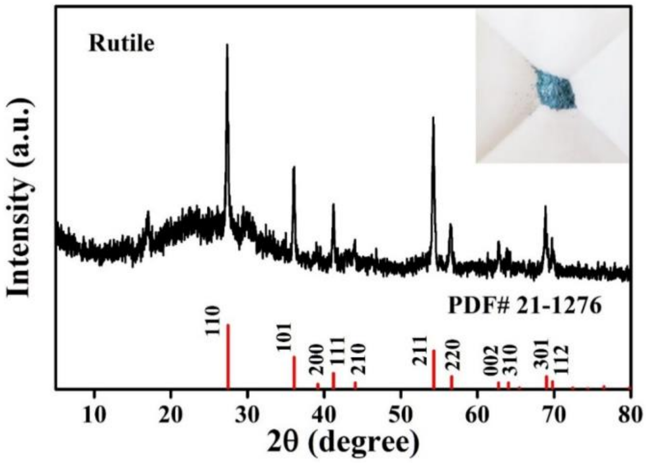

Fig. S13 XRD of the blue rutile-M-TiO_2 [S2] prepared by controlling the reaction conditions

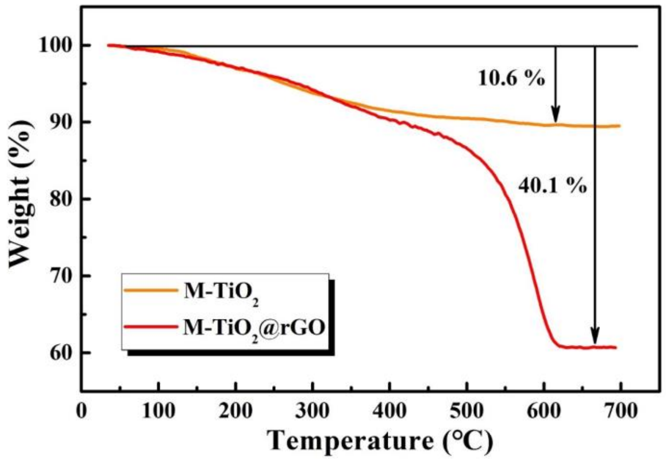

Fig. S14 TG curves of the M-TiO_2 and M-TiO_2@rGO

TiO_2 wt% $= \frac{1-40.1\%}{1-10.6\%} = 67\%$

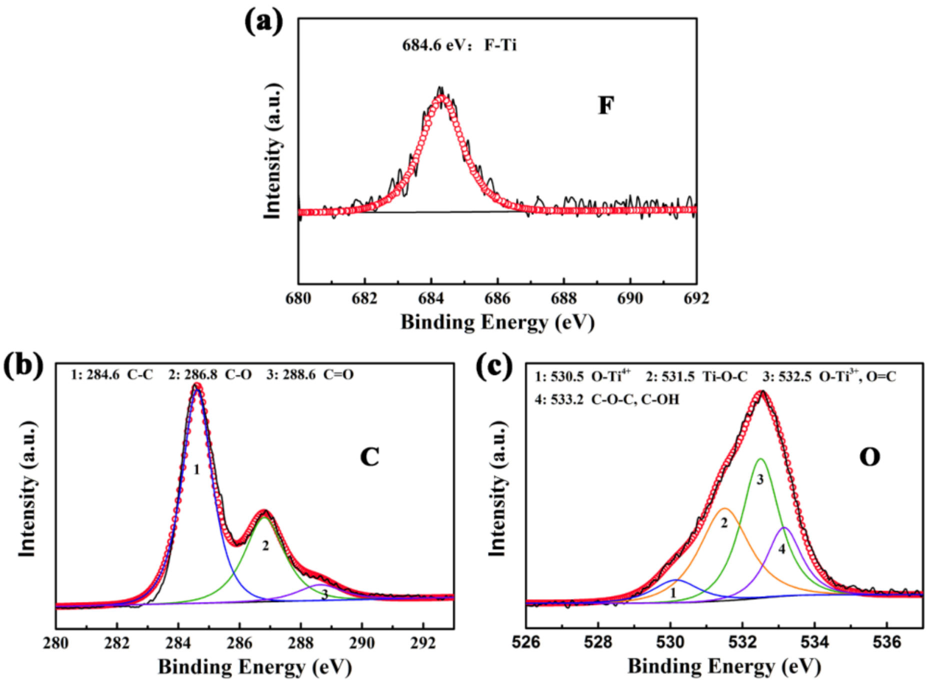

Fig. S15 XPS of M-TiO_2@rGO [S3-5]. (**a**) F 1s. (**b**) C 1s. (**c**) O 1s. From the **b** and **c** images, the M-TiO_2 links with rGO by Ti-O-C bond

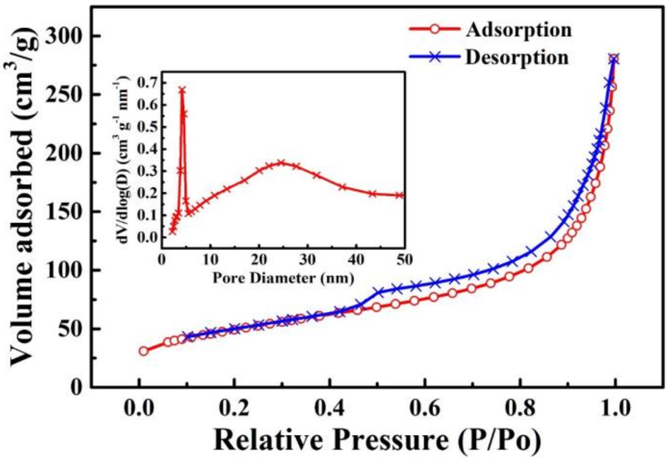

Fig. S16 BET curves of M-TiO_2@rGO. In inset: the pore size distribution

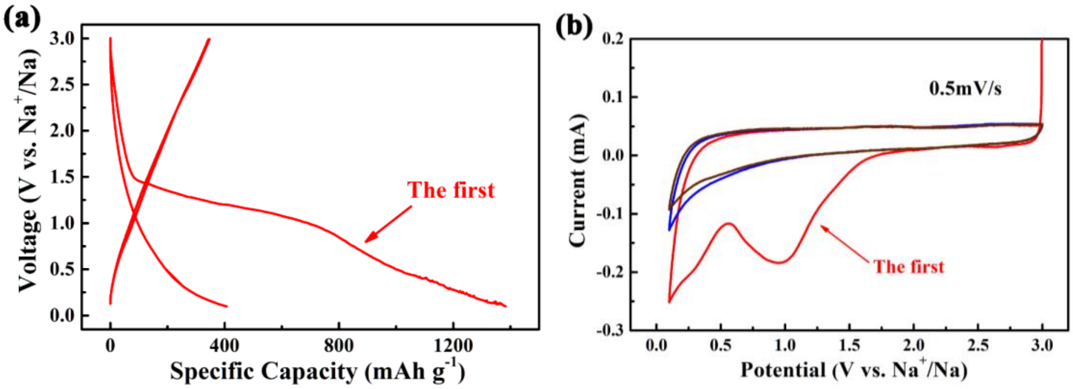

Fig. S17 Initial electrochemical performance of the M-TiO_2@rGO. (**a**) Charge-discharge curves at 50 mA g^{-1}. (**b**) CV curves at 0.5 mV s^{-1}. A large irreversible capacity was obtained below 1.0 V

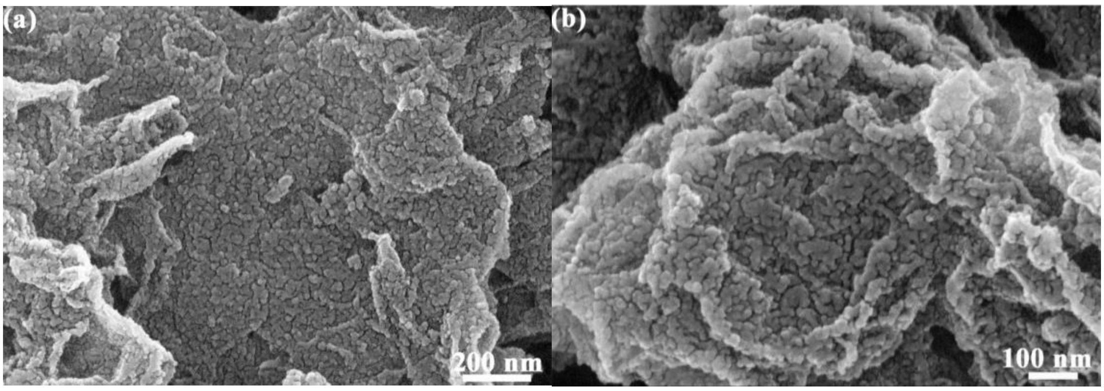

Fig. S18 SEM of the M-TiO_2@rGO electrodes after 1000 cycles at 1.0 A g^{-1}. (**a**) At low magnification. (**b**) At high magnification. The smooth edge of M-TiO_2 was attributed to repeated insertion and extraction of Na-ions

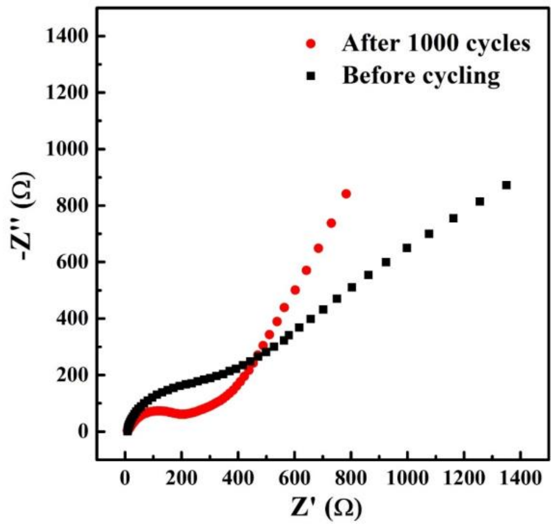

Fig. S19 EIS of the self-supporting M-TiO_2@rGO electrode before and after 1000 cycles at 1.0 A g^{-1}

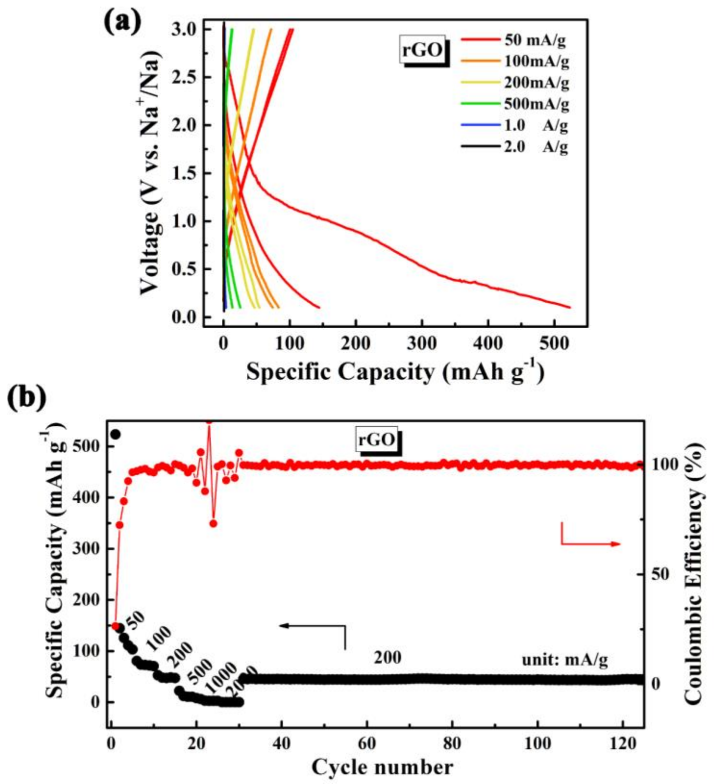

Fig. S20 Electrochemical performance of the rGO. (**a**) Charge-discharge curves at different current densities. A large irreversible capacity was observed at the first discharge. (**b**) Rate performance at different current densities and cycling performance at 200 mA g^{-1}

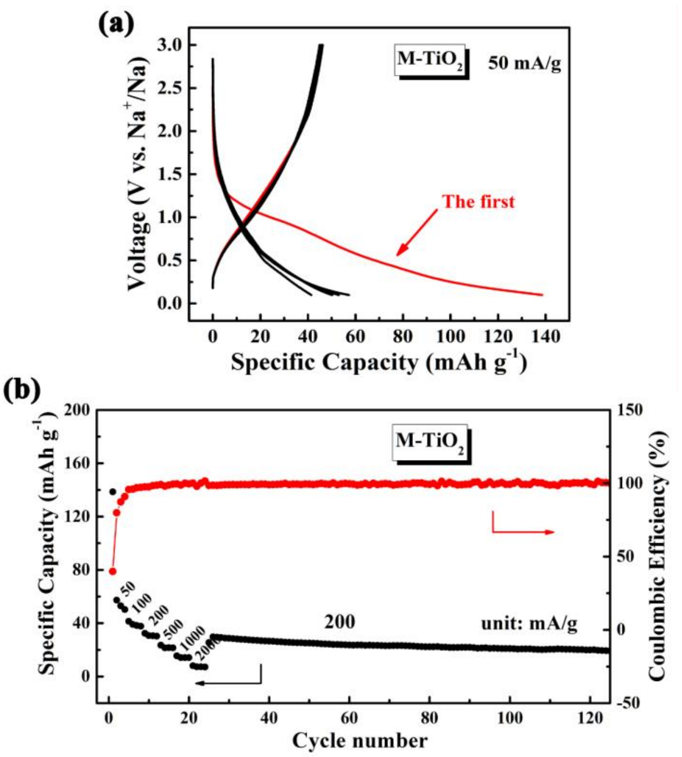

Fig. S21 Electrochemical performance of the M-TiO_2. (**a**) Charge-discharge curves at 50 mA g^{-1}. A large irreversible capacity was observed at the first discharge. (**b**) The rate performance with low capacity was obtained at different current densities and a cycling performance at 200 mA g^{-1}

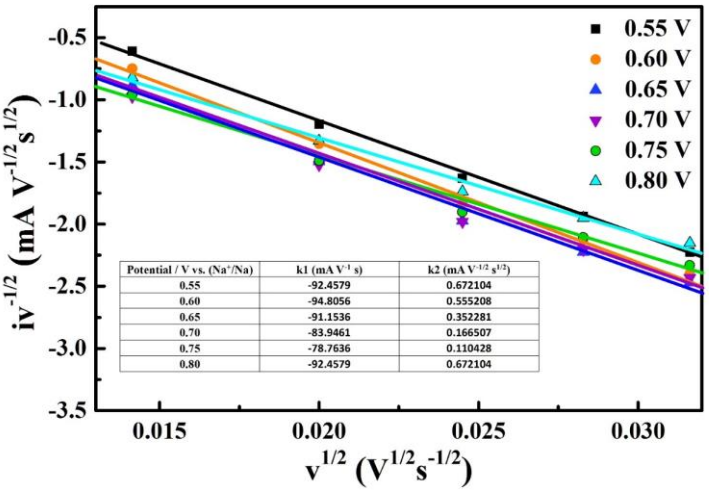

Potential / V vs. (Na^+/Na)	k1 (mA V^{-1} s)	k2 (mA $V^{-1/2}$ $s^{1/2}$)
0.55	-92.4579	0.672104
0.60	-94.8056	0.555208
0.65	-91.1536	0.352281
0.70	-83.9461	0.166507
0.75	-78.7636	0.110428
0.80	-92.4579	0.672104

Fig. S22 K values around the cathode peaks based on Fig. 5a

Calculation Method

$$I(\mathrm{V})=\mathrm{k}_1 v+\mathrm{k}_2 v^{1/2} \quad (2)$$

In Eq. (2), I-values are the current at a particular voltage and different scan rates, v is the scan rate, $k_1 v$ and $k_2 v^{1/2}$ represent surface and diffusion control, respectively [S1,

S6].

Converting the formula (2) to equation (3)

$$I(\mathrm{V})/v^{1/2}=\mathrm{k}_1 v^{1/2}+\mathrm{k}_2 \quad (3)$$

k_1 and k_2 can be obtained by finding the slope and intercept of $k_1v^{1/2}$ versus $I(\mathrm{V})/v^{1/2}$ at a specific potential and different sweep speeds as shown in Fig. S22. If the k_1 value at all potentials (or selection point) is found, the current value contributed by the capacitance can be obtained.

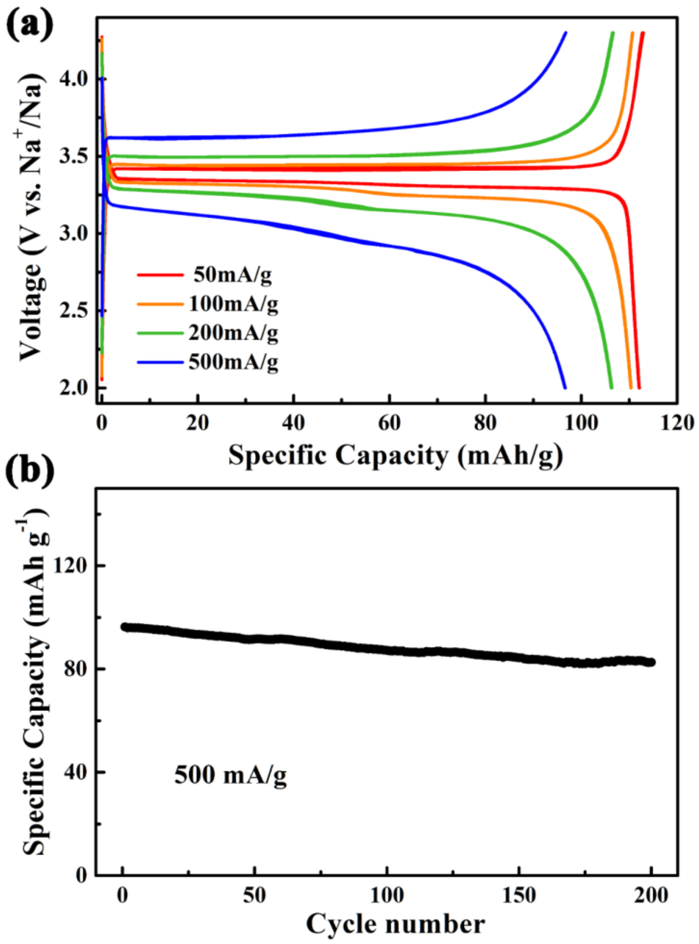

Fig. S23 Electrochemical performance of the NVP cathode in half-cells. (**a**) Charge-discharge curves. The specific capacities were 112.0, 110.4, 106.3, and 96.6 mAh g^{-1} at 50, 100, 200, and 500 mA g^{-1}. (**b**) Cycling performance at 500 mA g^{-1}. Capacity retention of 85% was obtained after 200 cycles

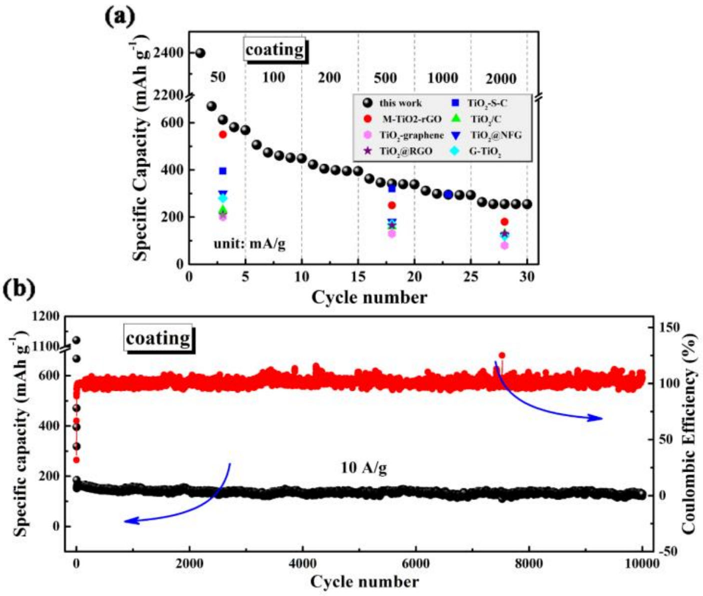

Fig. S24 Half-cells performance of the M-TiO_2@rGO electrodes coated on copper foil. (**a**) Rate performance at different current densities contrasted with other works, such as TiO_2-S-C [S7], M-TiO_2-rGO [S8], TiO_2/C [S9], TiO_2-graphene [S10], TiO_2@NFG [S11], TiO_2@RGO [S12], and G-TiO_2 [S6]. The coating-M-TiO_2@rGO electrodes exhibited average capacities of 610, 460, 400, 341, 295, and 255 mAh/g at 50, 100, 200, 500, 1000, and 2000, respectively. (**b**) Cycling performance at 10 A/g. An active process of 5 cycles was performed at 50 mA/g. After that, a capacity of 127.2 mAh/g and capacity retention of 84.6 % were obtained after 10,000 cycles

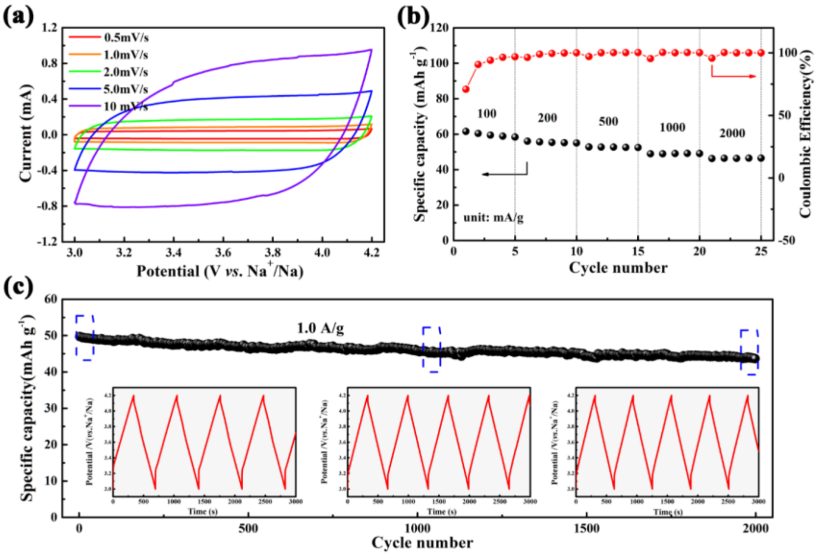

Fig. S25 Electrochemical performance of the HPAC in half-cells. (**a**) CV curves at different scan rates with little polarization phenomenon. (**b**) Rate performance at different current densities. The average specific capacities were 61.5, 55.2, 53.1, 49.6, and 46.4 mAh/g at 100, 200, 500, 1000, and 2000 mA/g, respectively. (**c**) A capacity retention of 89.3 % could be achieved after 2000 cycles at 1.0 A/g. The insets are the CP curves at different stages of cycling

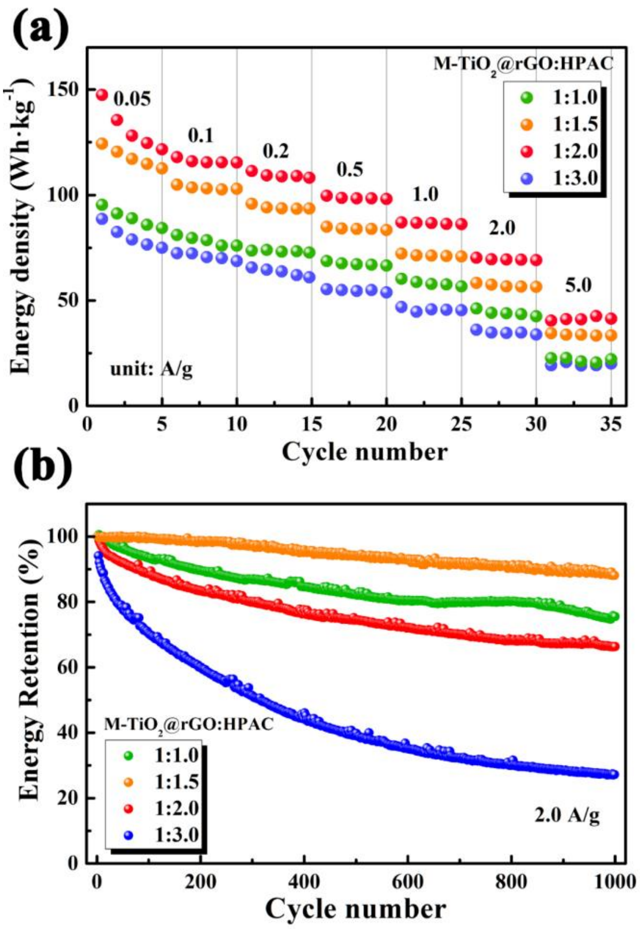

Fig. S26 Electrochemical performance of the SICs at different mass ratios. (**a**) Rate performance at different current densities. When the mass ratio was 1:2, the energy density was up to 147.3 Wh/kg (0.05 A/g), and after stabilization, it was 115.5 Wh/kg (0.1 A/g). When the mass ratio was 1:1.5, the energy density was up to 124.3 Wh/kg (0.05 A/g), and after stabilization, it was 101.2 W h/kg (0.1 A/g). The energy densities were lower at 1:1 and 1:3. (**b**) Cycling performance at 2.0 A/g. The energy retention ratios were 88.6, 75.5, 66.3, and 27.0 % at the mass ratio of 1:1.5, 1:1, 1:2, and 1:3

Supplementary References

[S1] Y. Fang, R. Hu, B. Liu, Y. Zhang, K. Zhu et al., Mxene-derived TiO_2/reduced graphene oxide composite with an enhanced capacitive capacity for li-ion and k-ion batteries. J. Mater. Chem. A **7**(10), 5363-5372 (2019). https://doi.org/10.1039/C8TA12069B

[S2] C. Gao, T. Wei, Y. Zhang, X. Song, Y. Huan et al., A photoresponsive rutile TiO_2 heterojunction with enhanced electron-hole separation for high-performance hydrogen evolution. Adv. Mater. **31**(8), 1806596 (2019). https://doi.org/10.1002/adma.201806596

[S3] H. Hu, Z. Zhao, W. Wan, Y. Gogotsi, J. Qiu, Ultralight and highly compressible graphene aerogels. Adv. Mater. **25**(15), 2219-2223 (2013). https://doi.org/10.1002/adma.201204530

[S4] B. Ahmed, D.H. Anjum, M.N. Hedhili, Y. Gogotsi, H.N. Alshareef, H_2O_2 assisted room temperature oxidation of Ti_2C MXene for Li-ion battery anodes. Nanoscale **8**(14), 7580-7587 (2016). https://doi.org/10.1039/C6NR00002A

[S5] C. Chen, Y. Wen, X. Hu, X. Ji, M. Yan et al., Na+ intercalation pseudocapacitance in graphene-coupled titanium oxide enabling ultra-fast sodium storage and long-term cycling. Nat. Commun. **6**, 6929 (2015). https://doi.org/10.1038/ncomms7929

[S6] Y. E. Zhu, L. Yang, J. Sheng, Y. Chen, H. Gu, J. Wei, Z. Zhou, Fast sodium storage in TiO_2@CNT@C nanorods for high-performance Na-ion capacitors. Adv. Energy Mater. **7**(22), 1701222 (2017). https://doi.org/10.1002/aenm.201701222

[S7] J. Li, X. Zhang, L. Han, D. Yan, S. Hou, T. Lu, Y. Yao, L. Pan, TiO_2 nanocrystals embedded in sulfur-doped porous carbon as high-performance and long-lasting anode materials for sodium-ion batteries. J. Mater. Chem. A **6**(47), 24224-24231 (2018). https://doi.org/10.1039/C8TA05617J

[S8] R. Wang, S. Wang, Y. Zhang, D. Jin, X. Tao, L. Zhang. Graphene-coupled Ti_3C_2 Mxenes-derived TiO_2 mesostructure: Promising sodium-ion capacitor anode with fast ion storage and long-term cycling. J. Mater. Chem. A **6**(3), 1017-1027 (2018). https://doi.org/10.1039/C7TA09153B

[S9] H. He, Q. Zhang, H. Wang, H. Zhang, J. Li et al., Defect-rich $TiO_{2-\delta}$ nanocrystals confined in a mooncake-shaped porous carbon matrix as an advanced Na ion battery anode. J. Power Sources **354**, 179-188 (2017). https://doi.org/10.1016/j.jpowsour.2017.04.035

[S10] Z. Le, F. Liu, P. Nie, X. Li, X. Liu et al., Pseudocapacitive sodium storage in mesoporous single-crystal-like TiO_2-graphene nanocomposite enables high-performance sodium-ion capacitors. ACS Nano **11**(3), 2952-2960 (2017). https://doi.org/10.1021/acsnano.6b08332

[S11] B. Li, B. Xi, Z. Feng, Y. Lin, J. Liu, J. Feng, Y. Qian, S. Xiong, Hierarchical porous nanosheets constructed by graphene-coated, interconnected TiO_2 nanoparticles for ultrafast sodium storage. Adv. Mater. **30**(10), 1705788 (2018). https://doi.org/10.1002/adma.201705788

[S12] Y. Liu, J. Liu, D. Bin, M. Hou, A. G. Tamirat, Y. Wang, Y. Xia, Ultrasmall TiO_2-coated reduced graphene oxide composite as a high-rate and long-cycle-life anode material for sodium-ion batteries. ACS Appl. Mater. Interfaces **10**(17), 14818-14826 (2018). https://doi.org/10.1021/acsami.8b03722
